# Aqueous proton transfer across single-layer graphene

**DOI:** 10.1038/ncomms7539

**Published:** 2015-03-17

**Authors:** Jennifer L. Achtyl, Raymond R. Unocic, Lijun Xu, Yu Cai, Muralikrishna Raju, Weiwei Zhang, Robert L. Sacci, Ivan V. Vlassiouk, Pasquale F. Fulvio, Panchapakesan Ganesh, David J. Wesolowski, Sheng Dai, Adri C. T. van Duin, Matthew Neurock, Franz M. Geiger

**Affiliations:** 1 Department of Chemistry, Northwestern University, 2145 Sheridan Road, Evanston, Illinois 60201, USA; 2 Center for Nanophase Materials Sciences, Oak Ridge National Laboratory, Oak Ridge, Tennessee 37831, USA; 3 Departments of Chemical Engineering and Chemistry, University of Virginia, 102 Engineers’ Way, Charlottesville, Virginia 22904-4741, USA; 4 Department of Chemical Engineering and Materials Science, University of Minnesota, 421 Washington Avenue SE, Minneapolis, Minnesota 55455, USA; 5 Department of Physics, Pennsylvania State University, University Park, Pennsylvania 16802, USA; 6 Department of Mechanical and Nuclear Engineering, Pennsylvania State University, University Park, Pennsylvania 16801, USA; 7 Measurement Science and Systems Engineering Division, Oak Ridge National Laboratory, Oak Ridge, Tennessee 37931, USA; 8 Department of Chemistry, University of Puerto Rico, Río Piedras Campus; San Juan, Puerto Rico 00931, USA; 9 Chemical Sciences Division, Oak Ridge National Laboratory, Oak Ridge, Tennessee 37831, USA

## Abstract

Proton transfer across single-layer graphene proceeds with large computed energy barriers and is therefore thought to be unfavourable at room temperature unless nanoscale holes or dopants are introduced, or a potential bias is applied. Here we subject single-layer graphene supported on fused silica to cycles of high and low pH, and show that protons transfer reversibly from the aqueous phase through the graphene to the other side where they undergo acid–base chemistry with the silica hydroxyl groups. After ruling out diffusion through macroscopic pinholes, the protons are found to transfer through rare, naturally occurring atomic defects. Computer simulations reveal low energy barriers of 0.61–0.75 eV for aqueous proton transfer across hydroxyl-terminated atomic defects that participate in a Grotthuss-type relay, while pyrylium-like ether terminations shut down proton exchange. Unfavourable energy barriers to helium and hydrogen transfer indicate the process is selective for aqueous protons.

## Abstract (web summary)

Proton transfer across graphene is associated with large computed energy barriers and is thought to be generally unfavourable. Here, the authors observe aqueous proton transfer through graphene subjected to pH cycling, suggesting that it is due to transfer through rare, naturally occurring atomic defects.

## Graphical abstract

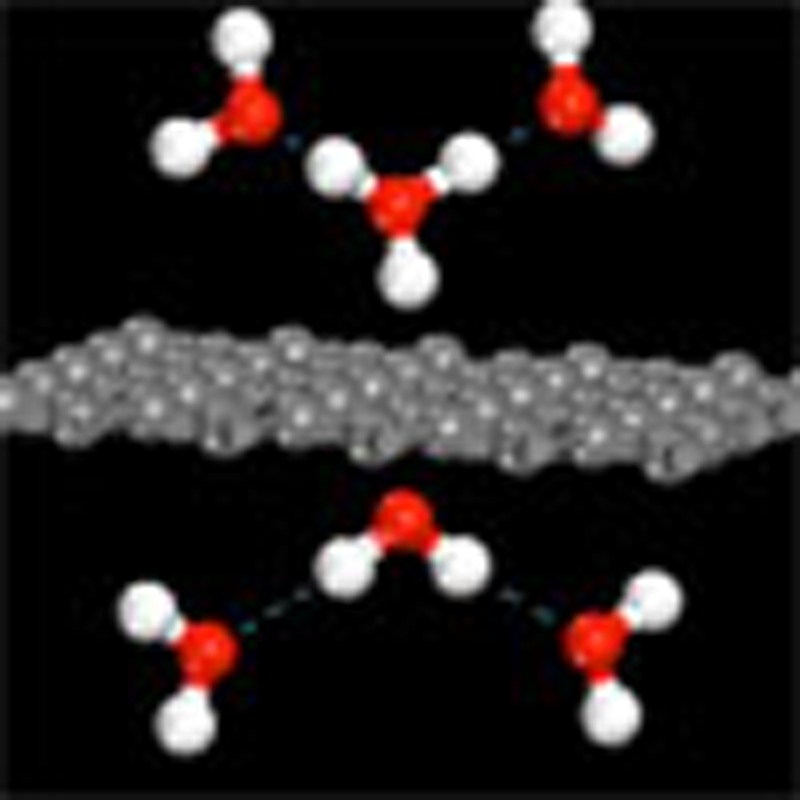

Brick-and-mortar networks of stacked graphene oxide nanosheets can act as effective membranes^1^,^2^,^3^,^4^,^5^,^6^,^7^,^8^, while single-layer graphene exhibits dramatically lower permeabilities towards gases^4^,^9^. In fact, graphene is thought to be unfit even for proton transfer, which is associated with computed gas-phase energy barriers exceeding 1.4 eV (ref. 10) unless dopants or nanoscale openings are externally introduced^6^,^7^,^10^,^11^, or an external potential bias is applied^12^. To determine whether graphene is indeed impermeable to protons, we place well-characterized single-layer graphene^13^ on top of a fused silica substrate and cycle, at room temperature and constant ionic strength, the bulk pH of an aqueous solution above the graphene layer between basic and acidic. We test for proton exchange through graphene by probing the underlying silica surface with an interfacial potential-dependent version of second harmonic generation (SHG)^14^,^15^ using 120 fs input pulses at energies well below the graphene damage threshold^13^. With a detection limit of 10^−5^ to 10^−6^ V (ref. 16), the method is sensitive enough to follow protonation or deprotonation of as little as 1% of the available silanol groups present in the area probed by SHG. The interfacial potential vanishes at the point of zero charge (PZC of fused silica ~2.5) (ref. 17), and the SHG signal intensity is small^14^,^18^,^19^. Increasing the pH at constant ionic strength shifts the relevant interfacial acid–base equilibria SiOH_2_^+^+OH^−^⇌SiOH+H_2_O and SiOH+OH^−^⇌SiO^−^+H_2_O (pKa ~4.5 and ~8.5)^14^,^18^,^20^ to the right, and the resulting interfacial potential polarizes the interfacial water molecules such that the SHG signal intensity increases^14^,^18^. Intuitively, the close proximity of the graphene layer and the charged fused silica surface, combined with the sensitivity of the method, make our approach akin to an Å-scale voltmeter for detecting even rare occurrences of proton exchange. We find no significant difference between the SHG versus time traces recorded in the presence and absence of graphene. After ruling out diffusion through macroscopic pinholes, the protons are found to transfer through rare, naturally occurring atomic defect sites in the graphene layer. Computer simulations reveal low energy processes for water-mediated proton transfer across hydroxyl-terminated atomic defect sites that participate in a Grotthuss-type relay, while defects terminated by pyrylium-like ether bridges shut down proton exchange.

## Results

### Silanol protonation and deprotonation unimpeded by graphene

**Figure 1:**
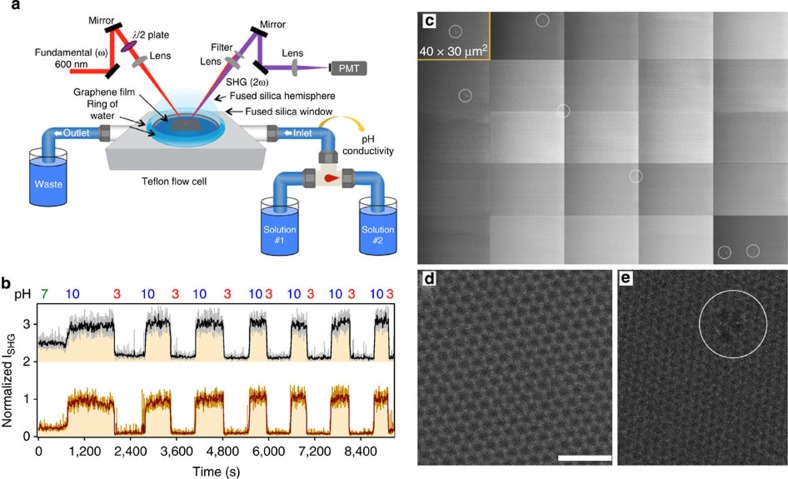
Experimental approach. (**a**) Experimental setup using a waveplate (*λ*/2) to prepare 600 nm light plane-polarized parallel to the plane of incidence (p-in) while a photomultiplier tube (PMT) detects the SHG photons at *λ*=300 nm. (**b**) p-in/all-out polarized SHG response recorded as a function of time from the fused silica/water interface during pH jumps from 7 to 3 to 10 and subsequent pH cycling between 3 and 10 at a bulk aqueous flow of 0.9 ml/s and 1 mM NaCl concentration in the absence (crimson, bottom) and presence (black, top, offset for clarity) of single-layer graphene placed between the fused silica substrate and the flowing bulk aqueous phase. Five-point boxcar indicated by dark lines. (**c**) Composite of 25 SEM images of single-layer graphene on a fused silica substrate, showing seven macroscopic pinholes, marked by white circles. (**d**) High-resolution aberration-corrected ADF STEM images of defect-free single-layer graphene on a transmission electron microscopy grid and (**e**) of a rarely imaged atomic defect. Scale, 1 nm.

Using a dual-pump flow system (Fig. 1a) at a flow rate of at ~0.9 ml s^−1^, we varied the bulk solution pH between 3 and 10 while maintaining constant 1 mM ionic strength (see Methods). As shown in Fig. 1b, we find no significant difference between the SHG versus time traces recorded in the presence and absence of graphene, and no statistically significant differences in the kinetic rates and jump durations (Supplementary Note 1, Supplementary Figs 1–2 and Supplementary Tables 1–2). The SHG responses to pH changes are consistent with the acid–base equilibria of the fused silica/water interface^14^,^15^,^19^,^21^, yielding effective pK_a,eff_ values of 3.5(1) and 8.3(2), which fall within the reported literature values (Supplementary Note 2 and Supplementary Fig. 3)^22^. This finding indicates that the SHG experiments do not track merely ion adsorption to the graphene/water interface but acid–base chemistry at the fused silica surface underneath it, for which proton transfer across the membrane is a necessary condition. As expected from refs 1, 2, 3, 4, 5, porous graphene multilayers do not inhibit proton transfer either (Supplementary Note 3 and Supplementary Figs 4–5). On the basis of these results we conclude that the fused silica/water interface does not behave differently in terms of relative surface charge density, in the duration of the jumps or in the rates of the jumps when single-layer graphene is present. These findings indicate that the acid–base chemistry at the fused silica/water interface occurs in an unimpeded fashion in the presence of single-layer graphene.

### Importance of macroscopic defects ruled out

Scanning electron microscopy (SEM, Methods) images of graphene single layers deposited on fused silica windows show a low density of macroscopic pinholes and that the graphene is free of cracks or folds (Fig. 1c). Two-dimensional (2D) diffusion from those locations to the location of the laser beam is considered by calculating, for a given proton diffusion coefficient *D*, the mean-square displacement, ‹Δ*r*^2^›, according to ‹Δ*r*^2^›=*z*·*t*·*D*, where *t* is time and *z* is the number of neighbouring sites to which the proton can hop^23^ (six in for the case of the hexagonal graphene lattice). In the literature, reported theoretically and experimentally determined proton surface diffusion coefficients range between 1.01 × 10^−7^ and 9.00 × 10^−5^ cm^2^ s^−1^ (refs 24, 25, 26, 27, 28, 29, 30, 31, 32, 33, 34, 35, 36, 37, 38, 39). While the bulk diffusion coefficient for a proton in water is accepted to range between 8 × 10^−5^ and 9 × 10^−5^ cm^2^ s^−1^, there are disagreements in the literature about whether the surface proton diffusion coefficient is similar to the bulk coefficient or slower than the bulk coefficient on hydrophobic and hydrophilic surfaces for a variety of different systems^24^,^28^,^38^. Reactivity is expected to substantially slow down the 2D diffusion of the proton (approximately magnitude × 20 reduction)^30^,^40^,^41^ when it moves across an amphoteric oxide whose protonation effectively terminates the diffusion path. Reactive proton diffusion coefficients reported for Nafion^42^,^43^ are similarly low. Indeed, our own reactive force field calculations containing partially hydroxylated quartz surfaces show the proton diffusion is quickly terminated by protonation of the surface SiO^−^ groups (Supplementary Note 4 and Supplementary Fig. 6). This result indicates that proton diffusion is significantly slower in the presence of surface anionic species due to proton trapping at these sites.

In our experiments, the continuous proton supply from the aqueous bulk is expected to form a propagating reaction front: our calculations show a drastically increased proton diffusion coefficient of 4.944 × 10^−5^ cm^2^ s^−1^, or just half of that of bulk water, once protons arriving through any opening within the graphene sheet interact with the hydroxylated portion of the surface that is located behind the reaction front. To conservatively assess an upper bound limit for our estimations, we calculated the proton mean-square displacement using a *D*-value of 1 × 10^−6^ cm^2^ s^−1^. The probability of placing our laser beam within the propagating reaction front emanating from a given macroscopic pinhole was then estimated to be 4% and 21% for 1 and 10 s SHG jump times, respectively (Supplementary Note 5, Supplementary Figs. 7–10 and Supplementary Table 3). Given that the pH jumps were repeated on at least 18 different days with eight different graphene samples and delays in changes of the SHG response were not observed with statistical significance; so we conclude that the diffusion of protons from the few macroscopic pinholes that are present in our samples, or, alternatively, from the sample edge, to the area probed by the laser cannot explain our observations of proton transfer through graphene.

### Imaging rare atomic defects

Scanning transmission electron microscopy (STEM) was then used to search for atomic defects using annular dark field (ADF) STEM imaging at 60 kV (see Methods). The majority of the images show perfect six-fold symmetry in the position of the carbon atoms and vast areas that lack grain boundaries and atomic, or vacancy, defects (Fig. 1d). Nevertheless, similar to prior reports of atomic-scale vacancy point defects^44^,^45^, we find, albeit rarely, atomic defects (Fig. 1e). Unless hydrocarbons or heavy metal atoms^46^ are present in graphene, defect formation due to electron beam-induced etching (as opposed to ion bombardment or oxidative etching)^47^ of pristine chemical vapour deposition (CVD) graphene at the energies employed here is unlikely. Rather, the rare defects we observe on occasion are more likely to originate from the synthesis process or cosmic rays, as the STEM experiments are carried out below the knock-on damage threshold for graphene^48^, and the femtosecond laser pulses are attenuated below the onset of processes other than SHG^13^. Given a lower limit to the estimated defect-to-defect distance of ~0.1 μm (ref. 49) (while difficult to determine accurately from Raman spectroscopy, the actual distance is likely to be longer), we assess the probability of placing our laser beam within the propagating reaction front emanating from a given atomic defect to be 100%.

## Discussion

**Figure 2:**
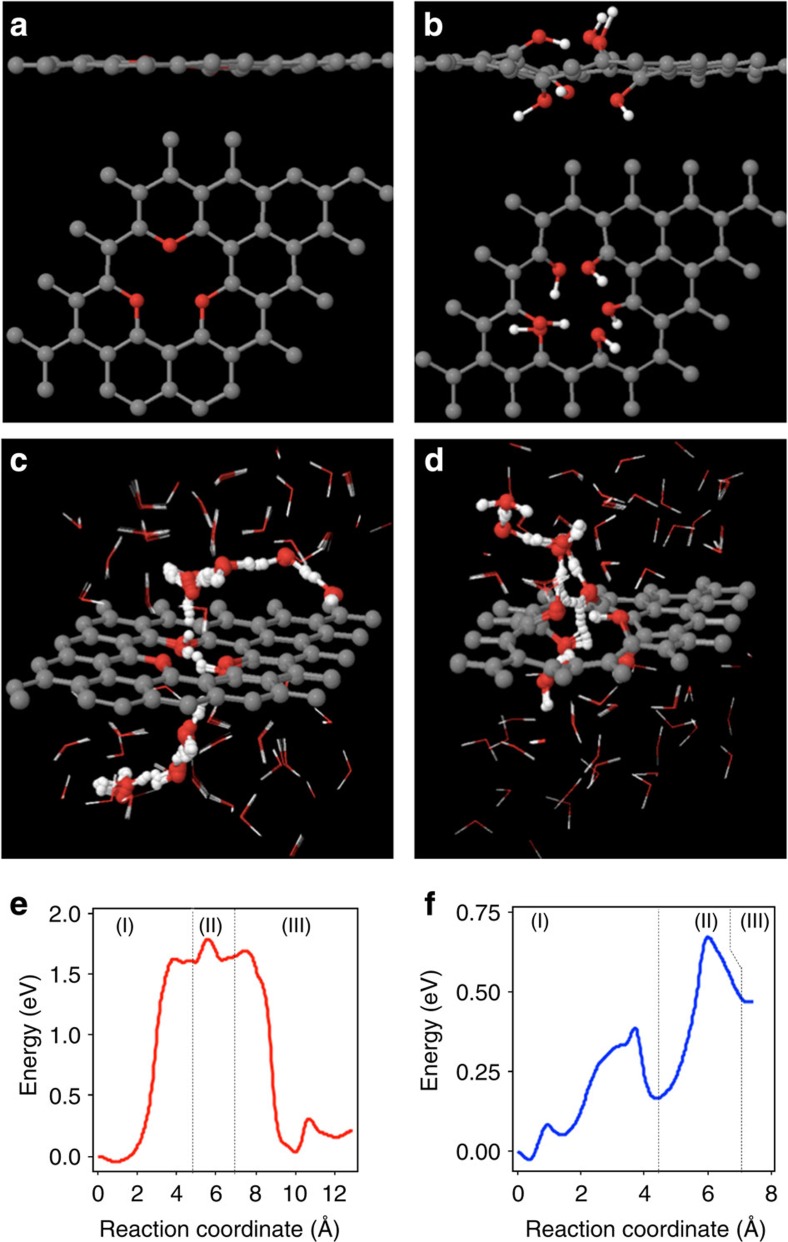
Density functional theory calculations. Side and top views of oxygen- (**a**) and OH- (**b**) terminated defect models used in the DFT calculations. Snapshots (**c**,**d**) and energetics (**e**,**f**) from the nudged elastic band calculations for proton transfer through the oxygen- and OH-terminated defect sites marking (region I) release of proton from H_3_O^+^ to oxygen and OH groups, respectively; (region II) relay of proton between oxygen and OH groups, respectively; (region III) release of proton from oxygen and OH groups to H_3_O^+^, respectively. Denotations of spheres: grey=carbon; red=oxygen; white=hydrogen atoms.

**Figure 3:**
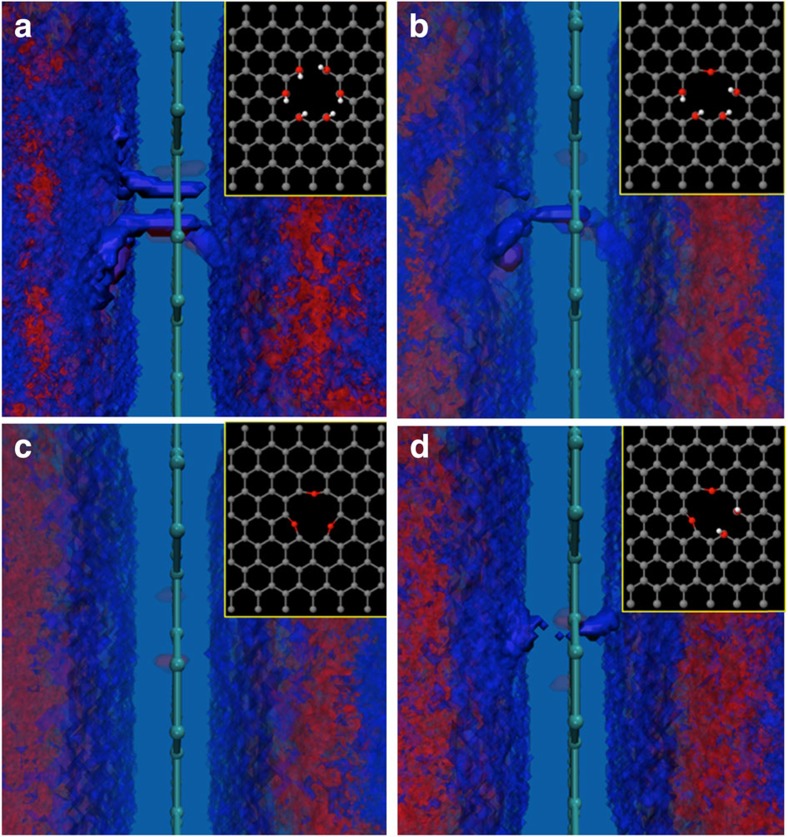
Reactive force field calculations. Proton channel formation from ReaxFF calculations of water-mediated proton transfer through atomic defects terminated in six OH groups (**a**), four OH groups and one oxygen atom (**b**), two OH groups and two oxygen atoms (**c**), and three oxygen atoms (**d**). Denotations of spheres: grey=carbon; red=oxygen; white=hydrogen atoms.

To elucidate the mechanisms for proton transfer, we discuss findings from density functional theory (DFT) calculations (Fig. 2) and ReaxFF reactive force field molecular dynamics (Fig. 3)^50^,^51^ simulations (Methods). DFT simulations track the detailed changes in the electronic structure and quantify the corresponding activation barriers as protons transfer from the water layer above the surface through the graphene interface and exit into solution on the opposite side of the surface. The ReaxFF simulations provide a larger scale representation of the interfaces and explicitly include dynamics.

We find that the main restriction for aqueous proton transfer through pristine, defect-free graphene is the energy required to push the proton through the center of an aromatic ring in the hydrophobic graphene layer as shown in Supplementary Fig. 11. While the protons readily migrate in the solution phase above and below the graphene surface via proton shuttling, they are unable to pass through the hydrophobic graphene layer. The energy costs to desolvate the proton from the aqueous layer and drive it through the center of an intact aromatic ring within the graphene layer are quite high and result in an activation barrier, that is, over 3.8 eV.

The basal planes of pristine graphene can, and do, contain rare atomic-scale defect sites comprised of carbon atom vacancies, as shown in Fig. 1e. Our calculations indicate that while the activation barrier for proton transfer through a single-vacancy site is over 1.9 eV lower than that for transfer through the pristine graphene surface, it is still nearly 2.0 eV due to the small size of the vacancy and the hydrophobicity of the surface. The formation of di- and tri-vacancy sites increases the diameter of the opening in the graphene layer and reduces the barrier further to ~1.5 eV, but this barrier is still too high to permit aqueous proton transfer at room temperature.

All of the defect terminations considered are energetically favourable as compared with the bare quad-vacancy system (Supplementary Note 6 and Supplementary Fig. 12). The removal of four carbon atoms in a central aromatic ring in the graphene layer leads to the formation of the quad-vacancy site as shown in Supplementary Fig. 13. This site is comprised of six coordinatively unsaturated carbon atoms that are either terminated with three oxygen atoms in epoxide-like arrangements reminiscent of pyrylium cations (different from the crown ethers recently reported by Guo *et al*.)^52^, or with six hydroxide groups. Proton transfer through the pyrylium-terminated quad-vacancy site requires 1.7 eV (Fig. 2a), attributed to the protophobicity of pyrylium cations and their in-plane localization, which leaves a 3.4 Å gap between water and the graphene substrate that prevents proton transfer. The hydroxyl-terminated site (Fig. 2b), however, provides hydrogen-bonding networks (Fig. 2d) that interconnect the graphene surface to the water layers above and below it. DFT and ReaxFF simulations indicate that these hydrogen-bonding networks serve as conduits that facilitate proton transfer from the solution phase to the surface through the center of the defect site and into the solution on the opposite side of the membrane via a Grotthuss mechanism^53^ involving proton shuttling. This proton transfer mechanism identified here involves relaying the proton from one of the top three defect hydroxyl groups to the next hydroxyl group and the next, subsequent transfer to one of the bottom three defect hydroxyl groups on the other side, and finally release into the aqueous phase. While solution-phase proton shuttling occurs with activation barriers <0.2 eV, the barrier for transferring the proton through the defect sites in graphene via the proton relay mechanism is just 0.68 eV (DFT, Fig. 2b, well reproduced by ReaxFF (0.61 eV)), indicating proton transfer will occur at room temperature.

Additional ReaxFF simulations show that a proton transfer channel, consisting o water molecules that transfer the protons through Grotthuss-type reactions, thins and finally vanishes when the pairs of OH groups terminating the defect site are successively replaced with oxygen atoms (Fig. 3a–d). These transfer paths are selective to aqueous protons as helium and H_2_ transfer requires barriers exceeding 1.9 eV (Supplementary Note 7 and Supplementary Fig. 14). Table 1 gives the comparison of activation barriers for proton transfer through graphene in water calculated by ReaxFF and DFT. The barriers given by DFT for the pristine and single-vacancy case are high (3.9 and >2.0 eV, respectively) and insurmountable during molecular dynamics (MD) simulations at 300 K. ReaxFF overpredicts the barriers for proton transfer in the pristine and single vacancy case. Yet, the barriers for the relevant quad-vacancy cases given by ReaxFF are in good agreement with DFT. Note that ReaxFF was not specifically trained against any of these barriers.

We conclude that aqueous protons transfer through single-layer graphene via rare, OH-terminated atomic defects at room temperature. While the rarity of the atomic defect sites would make it challenging to follow proton exchange across graphene using the pH-sensitive electrodes, the close proximity of the graphene layer and the charged fused silica surface, where the experimental observation of surface protonation and deprotonation is made by SHG, allows for the experimental observation of proton exchange across these rare defects. The associated energy barriers are comparable to recent experimentally determined activation energy barriers for proton transfer through graphene subjected to an externally applied potential^12^. From the SHG signal jump rates and the time required for 2D proton diffusion, we estimate that the presence of as few as a handful of atomic defects in a 1 μm^2^ area sample of single-layer graphene is sufficient to allow for the apparent unimpeded protonation and deprotonation of the interfacial silanol groups within 10 s (Supplementary Note 8 and Supplementary Fig. 15). Yet, we caution that given the limited accuracy with which the defect density can be determined in large (mm)-scale graphene, aqueous protons may transfer across single-layer graphene not only along the path discussed here but also along others as well. The identification of low barriers specifically for water-assisted transfer of protons through OH-terminated atomic defects in graphene, and high barriers for oxygen-terminated defects could be an important step towards the preparation of zero-crossover proton-selective membranes.

## Methods

### CVD graphene synthesis

We used graphene having a grain size of ~100 μm (ref. 54) grown on copper foils by atmospheric pressure CVD^54^,^55^. The graphene was transferred using spin coating of poly(methyl methacrylate) (PMMA) followed by copper etching in a FeCl_3_ solution and PMMA removal in acetone. The transfer was made onto clean fused silica substrates (ISP Optics, 1” diameter, QI-W-25-1, flatness 1 wave per inch at 633 nm) to fill ~1 cm^2^ with a single layer. Following annealing in a flow of 4% H_2_ in Ar for 30 min at 300 °C, vibrational sum-frequency generation spectra showed no evidence for CH stretches^56^. Similar to the finding of water layers between graphene and mica by atomic force microscopy^57^, there is probably water located between the graphene samples and the fused silica substrates used here.

### Scanning electron microscopy

SEM images were collected from the center of the graphene film. Graphene on an optical window was imaged using a Hitachi S-4800 scanning electron microscope (SEM) operating at 2 kV with a probing current of 10 μA and an Everhart–Thornley detector. Copper tape was used to reduce charging effects. Individual images were taken at × 1,200 magnification with 1,280 × 960 resolution. An array of 5 × 5 images (529 × 397 μm, pixel size 176 nm) was stitched together using Adobe Illustrator. Automatic brightness and contrast adjustment on each frame was carried out using the ‘auto adjust’ feature in Preview (Apple, Inc.). No other postedit feature or change was applied.

### Aberration-corrected scanning transmission electron microscopy

To confirm the single-layer nature of graphene synthesized using the CVD method^13^, atomic-resolution STEM imaging was performed at room temperature with an aberration-corrected Nion UltraSTEM-100 (ref. 58) equipped with a cold field-emission electron source. The microscope was operated at 60 kV, which is below the knock-on damage threshold for graphene. The CVD-prepared graphene specimens were transferred to a SiN-supported silicon microchip transmission electron microscopy grid. Before STEM imaging, the specimen was heated at 160 °C in vacuum (10^−5^ torr) for 8 h to remove surface contamination. Following heating in vacuum, the specimen was immediately transferred to the UltraSTEM for ADF STEM imaging. The surface of the graphene still retains residual PMMA that was used in the transfer processes to the transmission electron microscopy grid as shown in Supplementary Fig. 26; however, there are large areas that are devoid of the PMMA, which made it feasible to directly image the lattice structure and confirm the single-layer nature using atomic-resolution STEM imaging. The images were filtered using a smoothing function in Digital Micrograph, and the contrast and brightness were adjusted to enhance the contrast of the graphene.

### Aqueous solution and substrate preparation

The aqueous solutions were prepared with Millipore water, prepared the day before an experiment and left open to air overnight to equilibrate with atmospheric CO_2_ and NaCl (Alfa Aesar, 99+%). The concentration of NaCl was confirmed using a conductivity meter (Fisher Traceable Conductivity and TDS meter, Fisher Scientific). Solution pH was adjusted with minimum amounts of dilute solutions of ~1 M NaOH (Sigma-Aldrich, 99.99%) and HCl (EMD ACS grade). The pH-jump experiments were carried out using a fused silica hemisphere (ISP Optics, 1” diameter, QU-HS-25) pressed against either a fused silica window (ISP Optics, 1” diameter, QI-W-25-1) or a CVD-prepared graphene film transferred onto a silica window in an experimental setup previously reported^13^,^56^. The hemisphere and the fused silica window were cleaned before experiments by first treating the surface of interest with NoChromix (Godax Laboratories) for 1 h, rinsing with Millipore water and then storing in Millipore water overnight for SHG experiments the next day. On the day of the experiment, the bare fused silica window and hemisphere were sonicated in methanol for 6 min, dried in a 110 °C oven for 30 min, oxygen plasma cleaned (Harric Plasma) on high for 30 s, and then stored in Millipore water until the experiment. The graphene samples were not cleaned with this procedure, but were instead cleaned by flushing with ~2 l of Millipore water before each experiment. Supplementary Note 9 describes the graphene characterization and analysis by Raman and ultraviolet–visible spectroscopy (Supplementary Fig. 16) prior and after the pH-jump experiments.

### Flow system and flow cell

As shown in Fig. 1a, the graphene-on-fused silica sample or the silica window were clamped face down against a Viton O-ring on the Teflon flow cell^13^,^56^ so that the surface of interest was in contact with the aqueous phase. The fused silica hemisphere was then clamped on top of the window with a Millipore water layer in between in order to minimize the change of refractive index between the phases and to avoid the use of an index-matching fluid. Throughout the duration of the experiment, it was also necessary to maintain a ring of Millipore water around the bottom of the hemisphere in order to avoid evaporation of the sandwiched water layer. All of the experiments were completed with a 0.9 ml s^−1^ flow using variable flow peristaltic pumps as previously reported^13^,^56^. Using the flow system depicted in Fig. 1a, the pumps were switched to pull solutions from two different reservoirs. For the experiments reported here (excluding pKa experiments, see Supplementary Note 2), the two reservoirs contained 1 mM NaCl Millipore solutions adjusted to either pH 3 or 10. At the start and end of each pH-jump experiment, a 1 mM NaCl aqueous solution adjusted to pH 7 was pumped through the system, and the SHG signal was collected until it reached a steady state. It is assumed that the steady-state conditions were reached once the SHG signal remained at a stable intensity for a minimum of 300 s. After the system reached the steady state at pH 7, the flow was switched back and forth between the pH 3 and 10 aqueous solutions, each time allowing the SHG signal to reach steady state before switching to the next pH. After several pH 3 to 10 and pH 10 to 3 jumps were completed, the pH was adjusted back to pH 7, and the SHG signal was collected until the steady state was reached one last time. None of the liquid flow effects, reported for fused silica/water interfaces subjected to high shear rates^59^, were observed under the creeping flow conditions used here. Supplementary Note 10 and Supplementary Table 2 assess the flow dynamics in the cell.

### Laser and detection system

A detailed description of our SHG setup has been described previously^60^,^61^,^62^,^63^. Briefly, we use a regeneratively amplified Ti:sapphire system (Hurricane, Spectra Physics) that operates at a kHz repetition rate to produce 120 fs pulses to pump an optical parametric amplifier (OPA-CF, Spectra Physics) tuned to produce 600 nm light. After exiting the OPA, the beam is then directed through a variable density filter to attenuate the pulse energy to either 0.3±0.05 μJ per pulse for bare silica studies or 0.15±0.05 μJ per pulse for graphene studies. The pulse energy used for the graphene films equates to a power density of 2.1(7) × 10^4^ μJ cm^−2^ per pulse with a 30 μm focal spot, which is well below the damage threshold of graphene as previously reported^13^,^56^. At an angle just below total internal reflection, the p-polarized attenuated fundamental light is then directed through a fused silica hemisphere and focused at the graphene/water or silica/water interface. The reflected fundamental and second harmonic lights are directed through a Schott filter and a monochromator to remove any contributions at the fundamental frequency before amplification with a photomultiplier tube and detection using a gated single-photon counting system. Correct power dependencies and spectral responses are verified regularly, the SHG responses are well polarized, and sample damage does not occur^13^,^56^. Given that the SHG jump rates are independent of the mean stream velocity (Supplementary Note 1), we are confident that the acid–base reactions occurring at the fused silica surface are not mass transfer limited. Ultraviolet–visible and Raman spectra indicate that the samples are resistant to acid–base cycling under the conditions employed here.

### Computer simulations

First-principles periodic DFT calculations were carried out to determine the lowest energy interfacial water/graphene, water/graphene/water/silica structures and the activation barriers for proton diffusion through these interfaces using the Vienna *Ab Initio* Simulation Package^64^,^65^. In the DFT calculations, the reaction systems were modelled by optimizing a water phase above and below a single-graphene sheet. The simulations were carried out in a 5 × 5 supercell comprised of 50 carbon atoms, extended infinitively in the *x* and *y* dimensions. A 15 Å gap was inserted between the graphene layer perpendicular to the surface. The gap was subsequently filled with enough water molecules to match the overall density of water at 1.0 × 10^3^ kg m^−3^. The initial simulations were carried out with water on both sides of the graphene layer. The lower SiO_2_ substrate was initially simplified by using additional water. Subsequent calculations were carried out with more realistic slabs comprised of water/graphene/water/SiO_2_ substrates. The reaction rates and mechanisms of proton transfer through the graphene were described in the framework of transition state theory and within the harmonic approximation, which is robust for systems of high densities.

All of the calculations were carried out within the generalized gradient approximation using Perdew–Burke–Ernzerhof functional^66^ to treat exchange and correlation gradient corrections and projector augmented wave pseudopotentials^67^ to describe the electron–ion interactions. Plane wave basis sets with a cutoff energy of 400 eV were used to solve the Kohn–Sham equations for calculations for systems without water. Calculations for systems that include water solvation were carried out with cutoff energies for C and O of 283 eV. The surface Brillouin zone was sampled using a Monkhorst–Pack mesh of 3 × 3 × 1. All electronic energies were converged to within a tolerance of 1 × 10^−5^ eV. All of the atoms were allowed to relax in the geometry optimizations until the forces on each atom were <0.03 eV Å^−1^. Spin polarization was examined for all of the systems explored and applied when needed. Transition states were isolated using the nudged elastic band method^44^,^45^ together with the dimer method^68^. The nudged elastic band method was used to provide an initial transition state structure that was used in the subsequent dimer simulations to isolate the transition state. The reaction barrier was defined as the energy difference between the transition state and the reaction state minimum. The intrinsic barrier is defined as the energy gap between a transition state and its immediate reaction state. Given the importance of surface relaxation in atomically defected graphene layers^69^, all of our calculations on the single, di and quad carbon vacancy sites and the oxygen-terminated sites explicitly modeled surface relaxation (Supplementary Note 11 and Supplementary Figs 17–19).

The ReaxFF simulations were performed using the stand-alone ReaxFF implementation to study proton transfer through pristine graphene and graphene with di and quad vacancies. We then compared with results from long *ab initio* MD to validate predictions of force field in describing water/graphene systems (Supplementary Note 12 and Supplementary Fig. 20). In our simulations, we used a (6 × 6) periodic graphene sheet with water molecules placed in random configurations on either side of the graphene sheet. The dimensions of the simulation cell are 15.01 × 17.83 Å parallel to the sheet and 30 Å in the direction perpendicular to the sheet. All MD simulations have been performed in the canonical (NVT, constant number of atoms (N), constant volume (V) and constant temperature (T)) ensemble, with a time step of 0.25 fs using the Berendsen thermostat with a coupling time constant of 100 fs to control temperature of the entire system. To obtain the density plots in Fig. 3, we first divided the simulation cell into a mesh of cubic boxes with dimensions (0.30 × 0.30 × 0.30 Å). We then counted the number of times a particular atom type (for example, oxygen) was located in each of the grids through the entire length of simulation and normalized these numbers by the highest count recorded in any of the grids. We used these normalized values to obtain the resulting density plots in Fig. 3. The ReaxFF results reproduce the DFT results, described well in further detail in Supplementary Note 13 and Supplementary Figs 13 and 21–25.

## Author contributions

F.M.G. conceived of the idea; J.L.A., R.R.U., R.L.S., I.V.V. and P.F.F. performed the experiments; L.X., Y.C., M.R., W.Z., P.G., A.C.T.v.D. and M.N. performed the computational work. The manuscript was written with substantial contributions from all authors.

## Additional information

**How to cite this article**: Achtyl, J.L. *et al*. Aqueous proton transfer across single-layer graphene. *Nat. Commun*. 6:6539 doi: 10.1038/ncomms7539 (2015).

## Supplementary Material

Supplementary Information Supplementary Figures 1-26, Supplementary Tables 1-3, Supplementary Notes 1-13 and Supplementary References

## Figures and Tables

**Table 1:** DFT and ReaxFF-calculated activation barriers for proton transfer through different vacancy sites on graphene in water.

Graphene surface (number of vacancies)	Bottom layer	Defect termination	Activation barrier DFT (eV)*	Activation barrier ReaxFF*
None	Water	No termination	3.9	Not computed
1	Water	No termination	>2.0	3.54 eV
4	Water	No termination	0.25	0.22 eV
4	Water	3O ether capped	1.8	1.7 eV
4	Water	6 OH hydroxyl capped	0.68	0.61 eV
4	Water+SiO_2_	3 O ether capped	2.5	2.53 eV
4	Water+SiO_2_	6 OH hydroxyl capped	0.7	0.75 eV

DFT, density functional theory.

^*^The energy difference that is reported is due to the initial reference (or reactant) state.
