# Supplementary material for: Aqueous proton transfer across single-layer graphene
Source: Nat Commun. 2015 Mar 17;6:6539. doi: 10.1038/ncomms7539 (PMC4382684; doi:10.1038/ncomms7539)
Supplement: Supplementary Information — Supplementary Figures 1-26, Supplementary Tables 1-3, Supplementary Notes 1-13 and Supplementary References [file ncomms7539-s1.pdf]

Supplementary figures

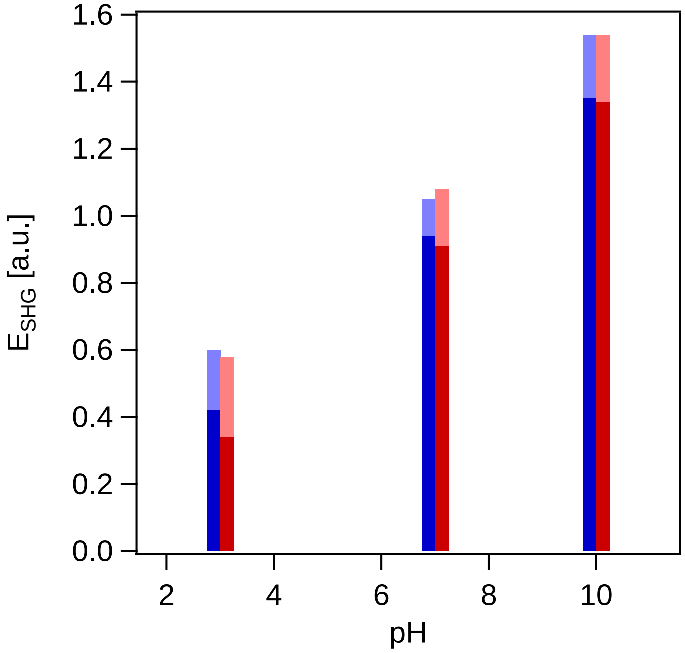

Supplementary Fig. 1 | pH-dependent SHG E-fields from fused silica with and without graphene. Comparison of the average SHG E-fields from pH 3, pH 7, and pH 10 adjusted 1 mM NaCl Millipore solutions over a single layer graphene film (blue bars) and a bare fused silica window (red bars). The resulting SHG E-fields were normalized to the averaged E-field calculated from the SHG intensities collected from a pH 7 solution at the beginning and end of each "pH jump experiment". Lighter red and blue colors represent the ± uncertainties (1σ) of the point estimates.

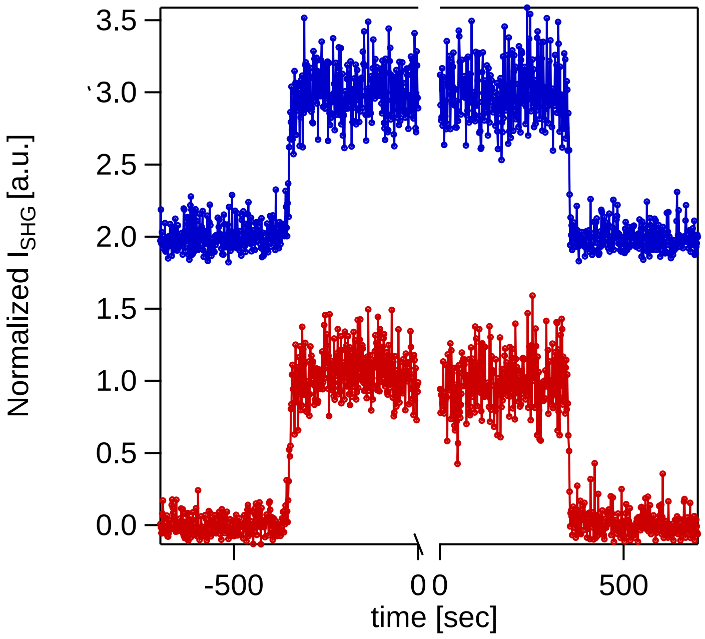

Supplementary Fig. 2 | SHG vs time traces. A comparison of the duration of the 3 to 10 pH jumps (left) and 10 to 3 pH jumps (right) for the single layer graphene film (blue traces) and the bare fused silica (red traces) at a 0.3 mL/sec flow rate. Each pH jump time trace is referenced to pH 3 and normalized to pH 10 and is the average of 4 individual SHG pH jumps. The different SHG traces were averaged together centering each jump such that the calculated inflection points were centered at the same time value. The graphene traces are offset for clarity.

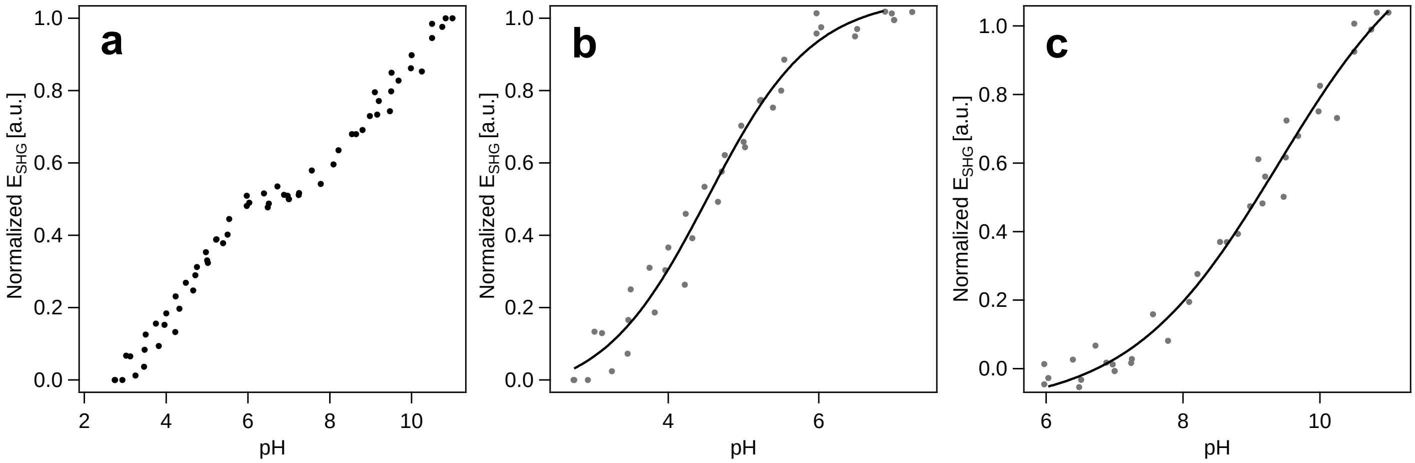

Supplementary Fig. 3 | Interfacial titration curves by SHG. The normalized SHG E-field plotted as a function of pH from the water/single layer graphene/fused silica interface in the presence of 100 mM NaCl. The data is the compilation of 3 separate experiments run on 2 different graphene samples. The E-field values from each day are normalized to the averaged E-fields obtained from pH 7 aqueous solutions. **(a)** The SHG E-fields referenced to the minimum E-field and normalized to the maximum E-field, showing the two inflection points consistent with the bimodal acid-base equilibria of the fused silica interface. **(b)** The SHG E-fields referenced to the E-field value at pH 2.75 and normalized to E-field value at pH 7.25. The line represents the sigmoid fit of the data. The inflection point here was used to calculate the pKa^{eff} of the more acidic silanol groups. **(c)** The SHG E-fields referenced to the E-field value at pH 6 and normalized to E-field value at pH 11. The line represents the sigmoid fit of the data. The inflection point here was used to calculate the pKa^{eff} of the less acidic silanol groups.

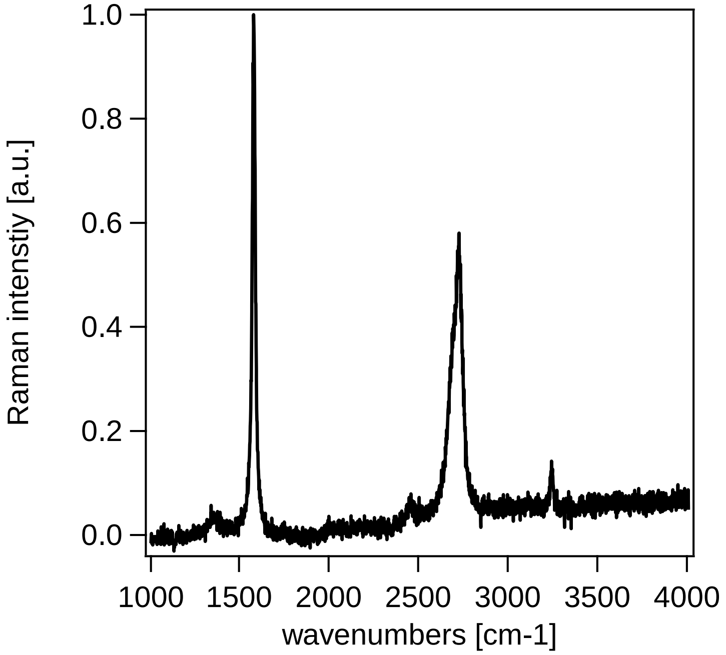

Supplementary Fig. 4 | Raman spectra of multilayer graphene. A representative Raman spectrum obtained from the multilayer graphene sample with the characteristic 2D, G, and D bands indicative of multilayer graphene films. The spectrum is normalized to the maximum Raman intensity value.

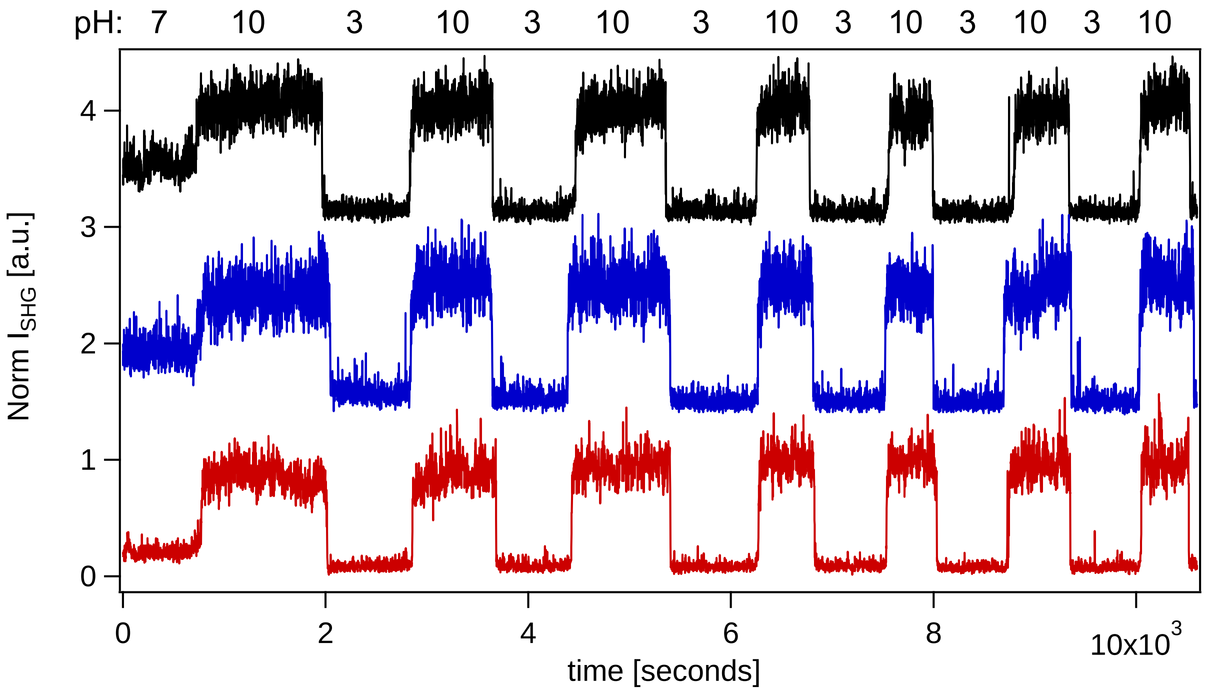

Supplementary Fig. 5 | SHG vs time traces. Normalized SHG intensity recorded as a function of time from the water/bare fused silica (crimson trace), water/single layer graphene/fused silica (blue trace) and water/multilayer graphene/fused silica (black trace) interfaces during "pH jumps experiments". The traces were recorded with a 0.9 mL/sec flow rate starting at pH 7 and then cycling between pH 10 and 3 with a 1mM NaCl Millipore water solution.

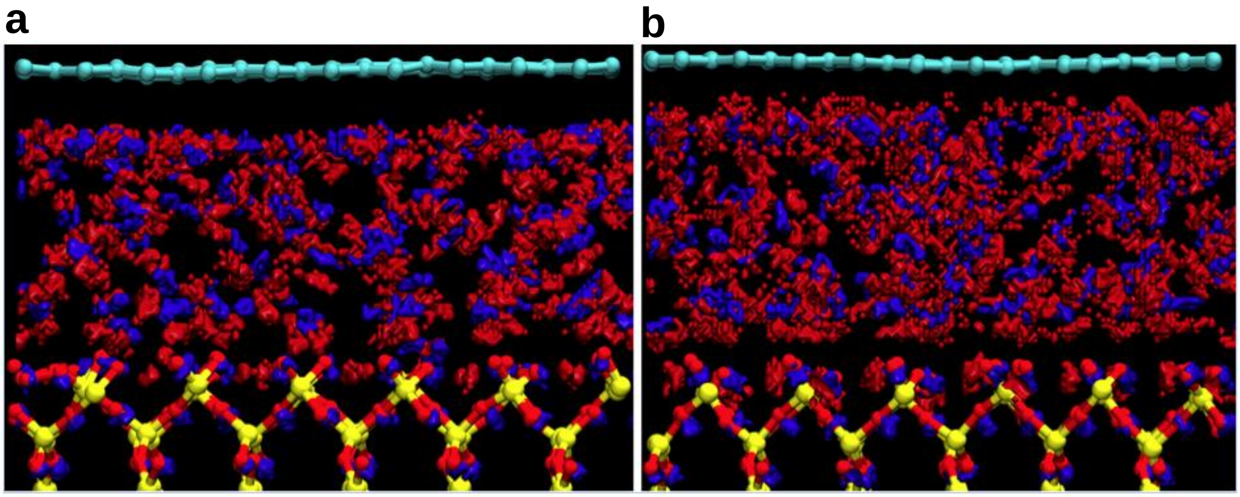

Supplementary Fig. 6 | Water structure between SiO_2 and graphene. Density plot of oxygen(blue) and hydrogen(red) in the silica/water/graphene interface, as obtained during a 300K ReaxFF molecular dynamics simulation. (**a**) structure at high pH (40% SiOH/60% SiO^-; OH-ions in water solution) – showing very strong local water structure and 20-fold reduced water self diffusion (**b**) water structure at neutral/low pH (100% SiOH; pure H_2O) showing more liquid water structure and water self-diffusion comparable to bulk water. Denotations of spheres: yellow=silicon; red=oxygen; cyan=carbon atoms.

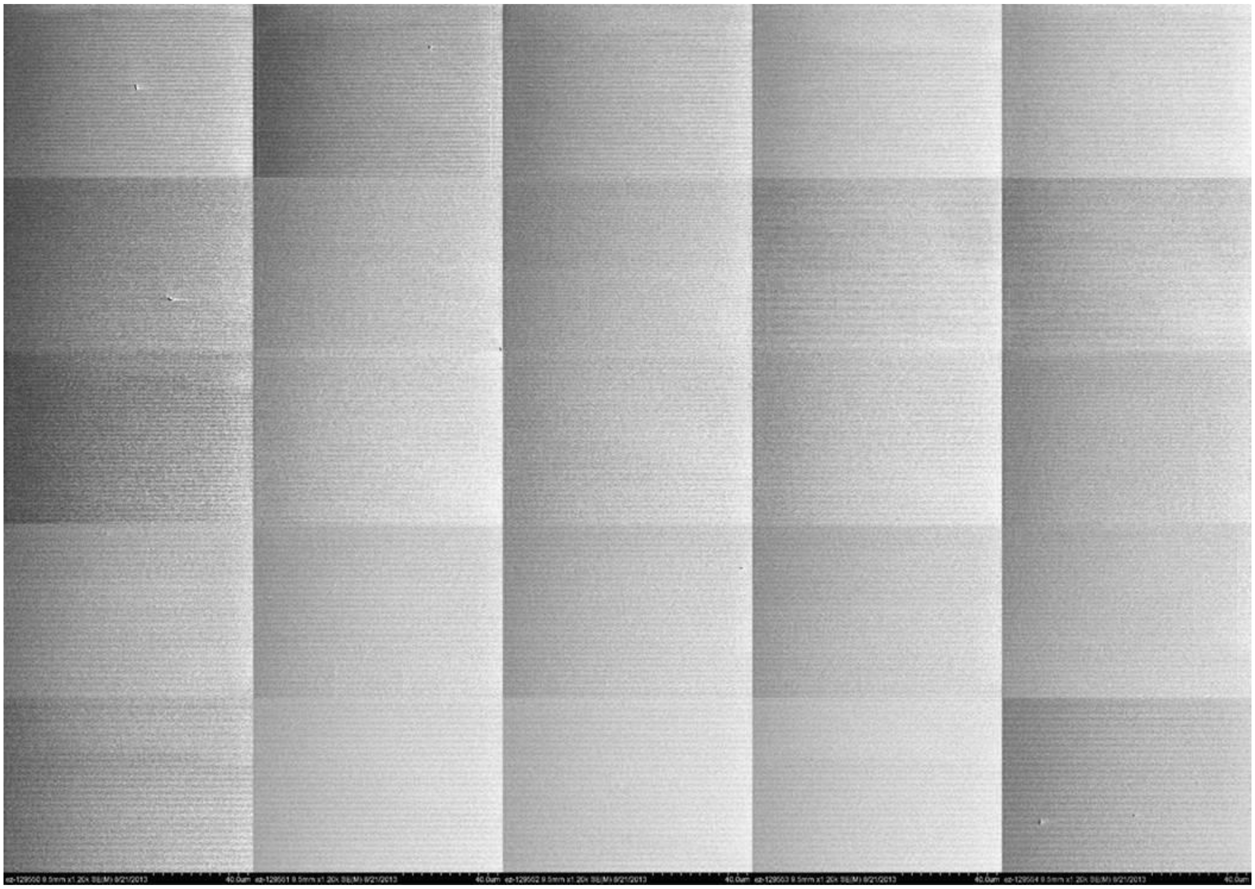

Supplementary Fig. 7 | Imaging macroscopic defects by SEM. Resulting image from combining the SEM images collected over a 529 µm x 397 µm area at the center of the graphene film.

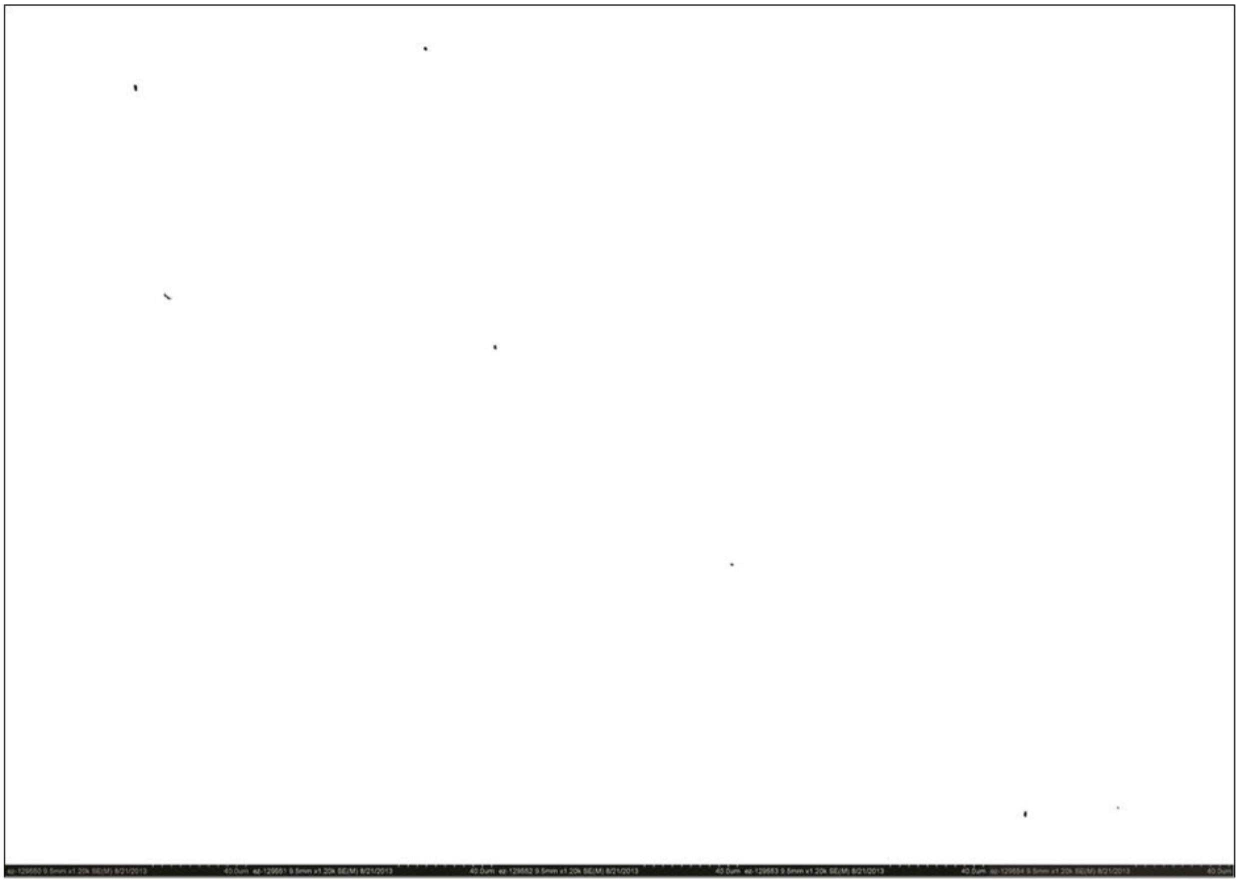

Supplementary Fig. 8 | Locating macroscopic defects. Th**e** resulting “masks” image returned from running the ImageJ “Analyze Particles” program after thresholding on the pinhole-marked SEM image as described in the Supporting Information text. The dark features represent the detected anomalies larger than 9 pixels in size. The 7 anomalies shown here were all counted and treated as pinholes.

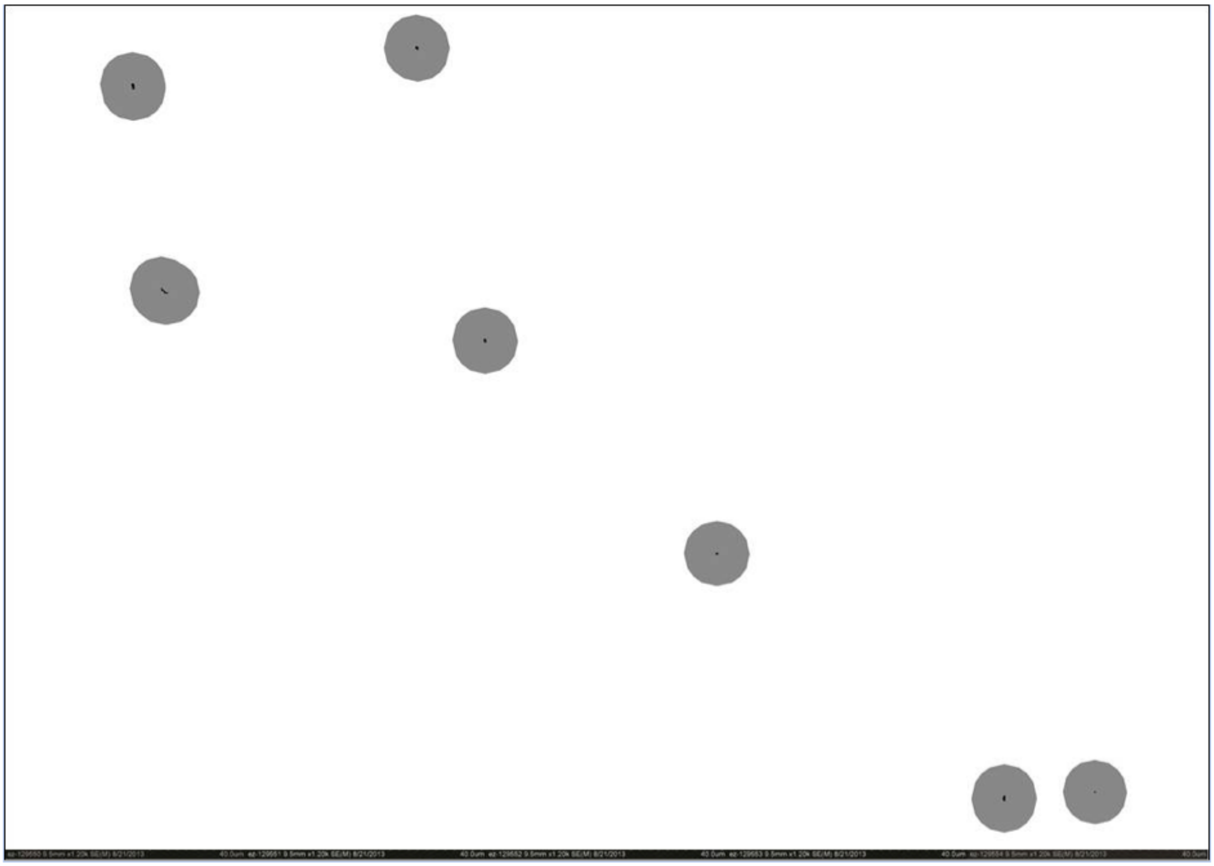

Supplementary Fig. 9 | 2D-diffusion calculation for macroscopic defects I. The resulting image after increasing the size of the pinholes (black) on all sides by the proton's radius of diffusion (grey) calculated for 1 second duration and a 1 x 1 x 10^{-6} cm^2/sec D value. The "diffusion detection area", resented by the black and grey features covers 1.9 % of the total image area.

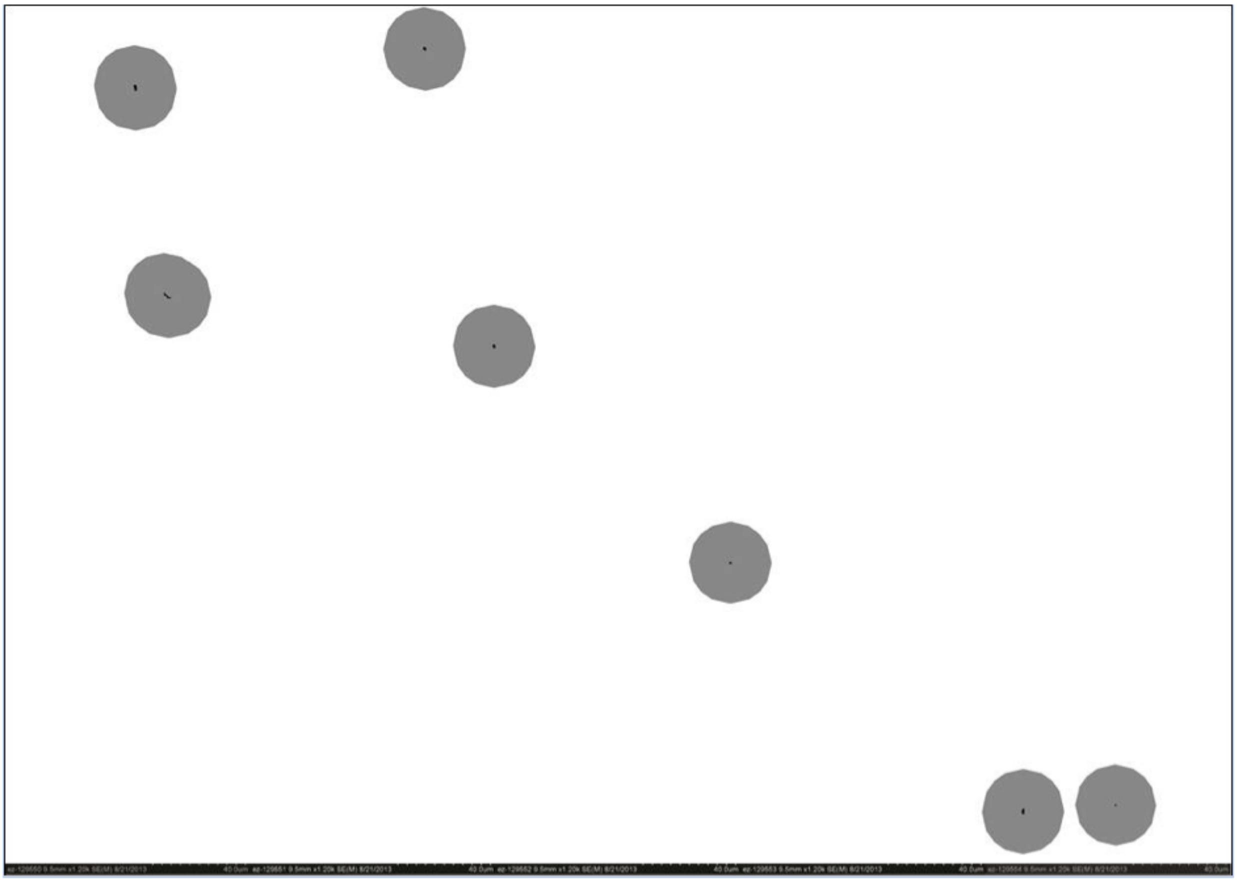

Supplementary Fig. 10 | 2D-diffusion calculation for macroscopic defects II. The resulting image after increasing the size of the pinholes (black) on all sides by the proton's radius of diffusion (calculated for a 1 second duration and a 1×10^{-6} cm^2/sec D value) and the 3μm distance to account for the partial overlap of the laser spot with the "diffusion detection area" (grey). This resulting "diffusion detection/laser spot area", resented by the black and grey features, covers 2.9 % of the total image area.

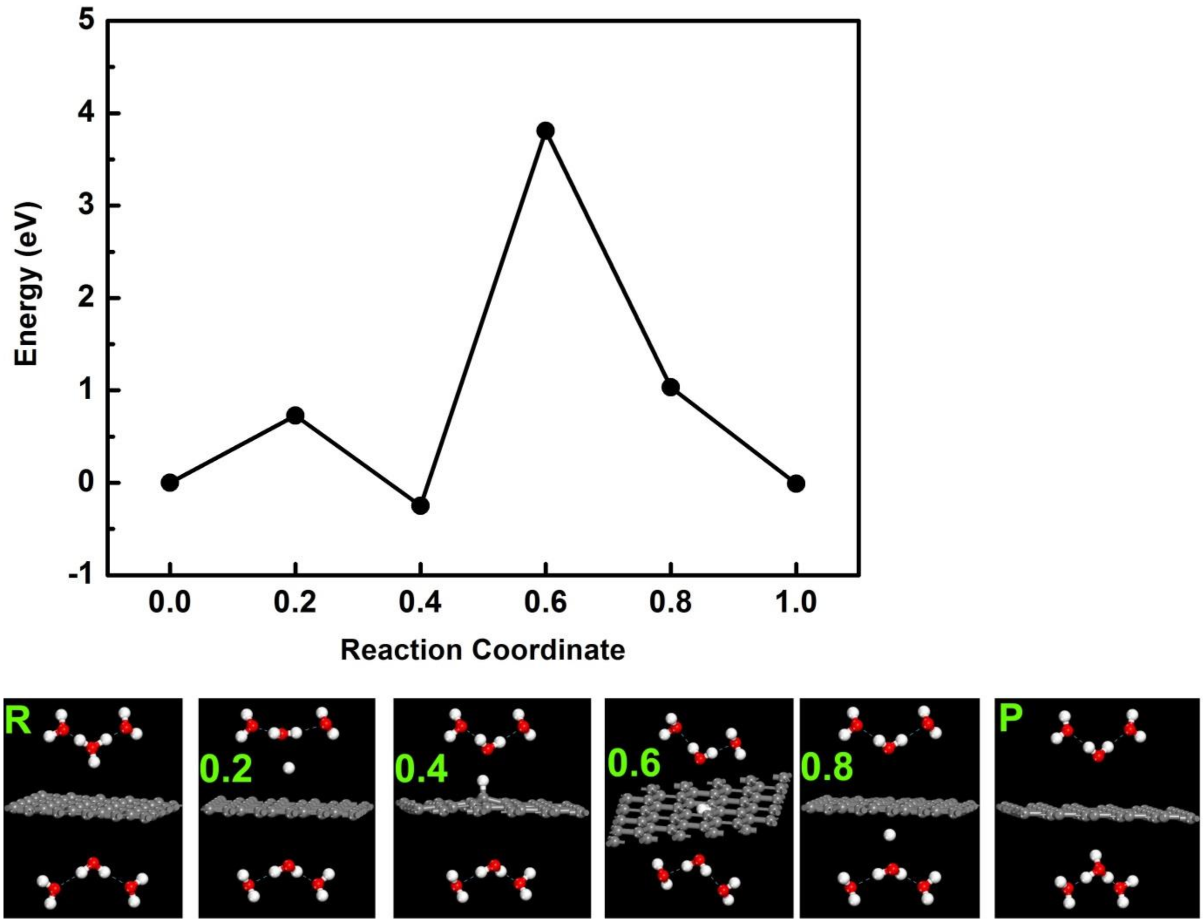

Supplementary Fig. 11 | Aqueous Proton transfer through pristine graphene. Energy barrier diagram and snapshots from nudged elastic band (NEB) calculations from reactant state "R" to product state "P" in fractional steps of 0.2.

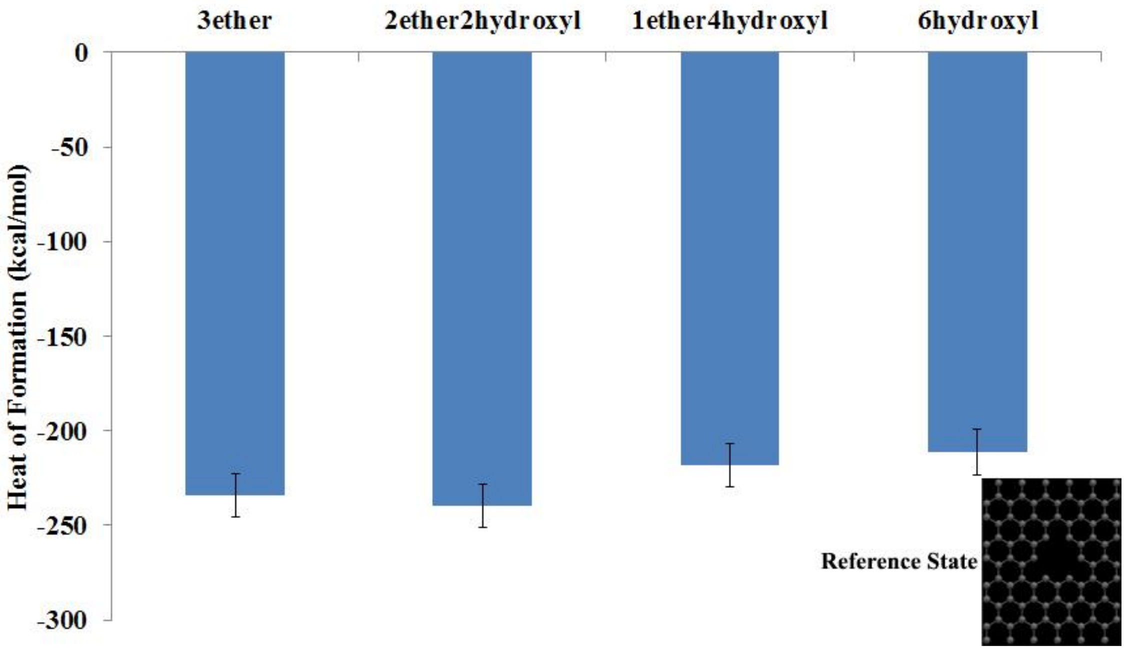

Supplementary Fig. 12 | Stability of defect terminations. Heats of formation for various defect terminations on quad-vacancy graphene.

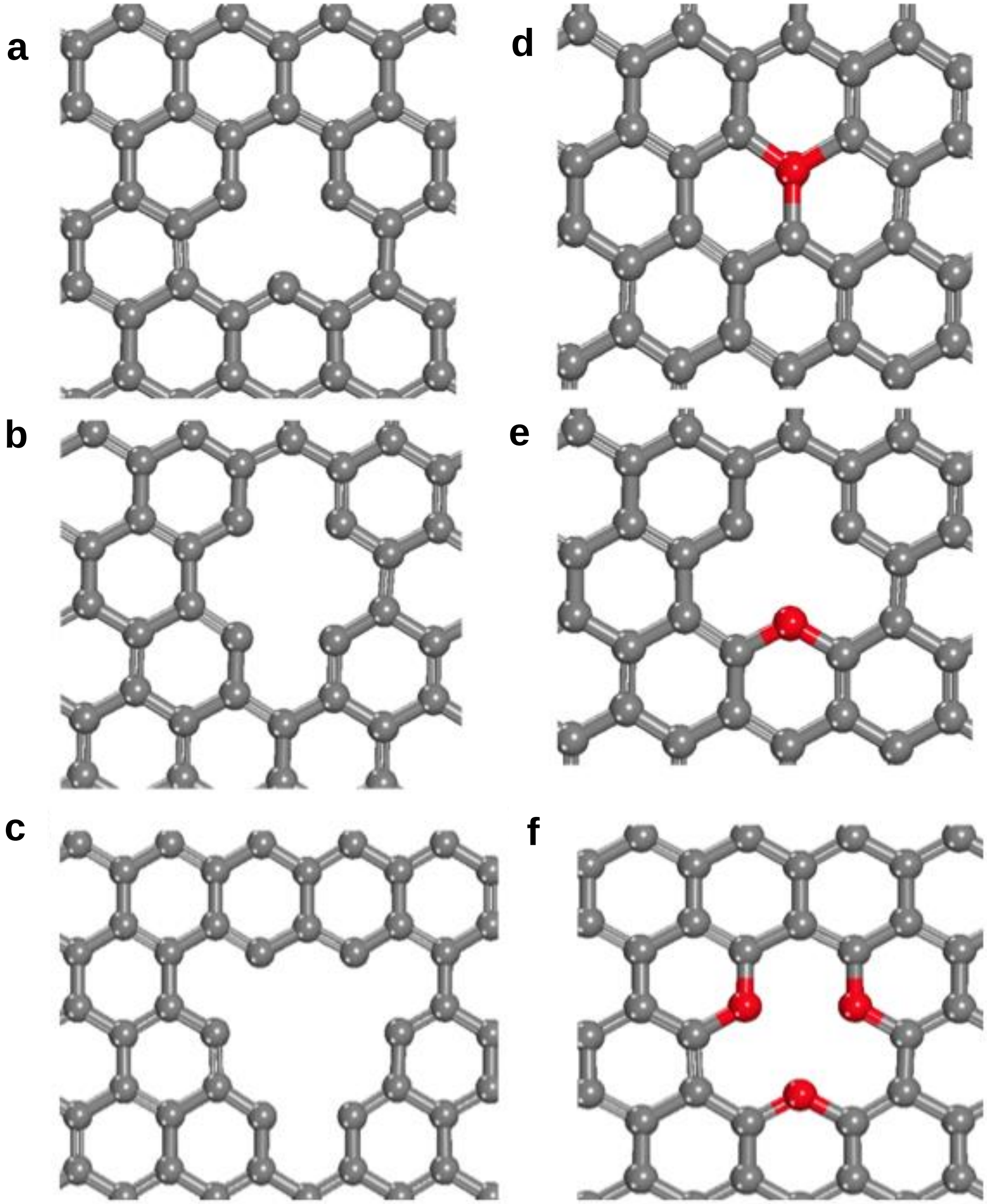

Supplementary Fig. 13 | Atomistic views of some of the coordinatively unsaturated defect sites considered here. The 1, 2, 4 (4V) carbon vacancy sites and the oxygen terminated vacancy sites in graphene.

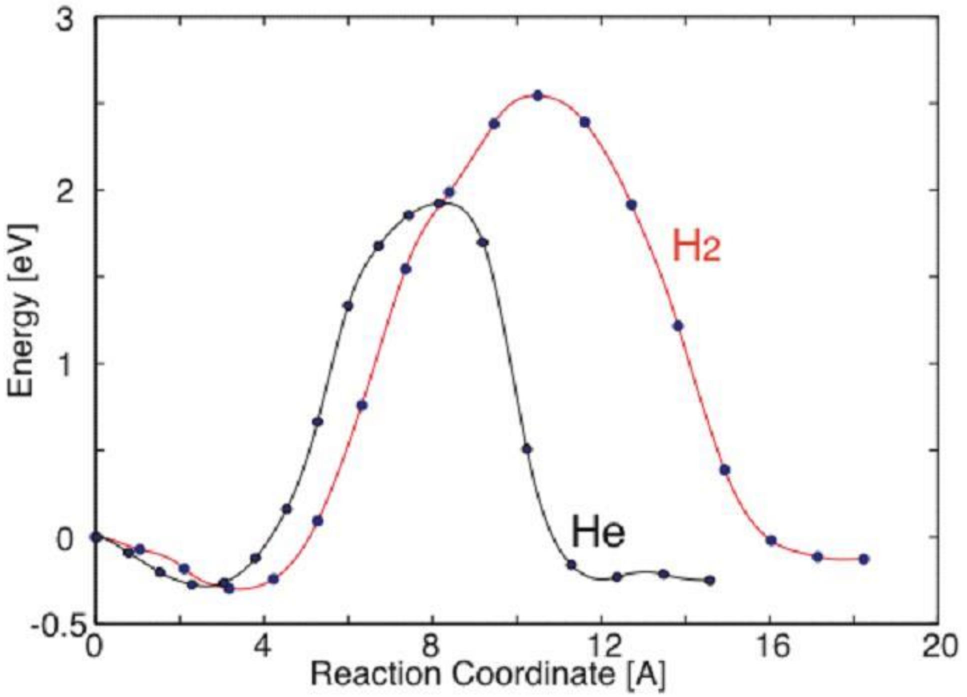

Supplementary Fig. 14 | He and H_2 transfer across OH-terminated defect site. DFT-calculated energetics for He and H_2 transfer through hydroxyl-decorated graphene indicate the barrier for He diffusion is 1.8 eV while that for H_2 is over 2.5 eV through the OH-terminated 4V site, which indicates that neither of these species would transfer through graphene at room temperature.

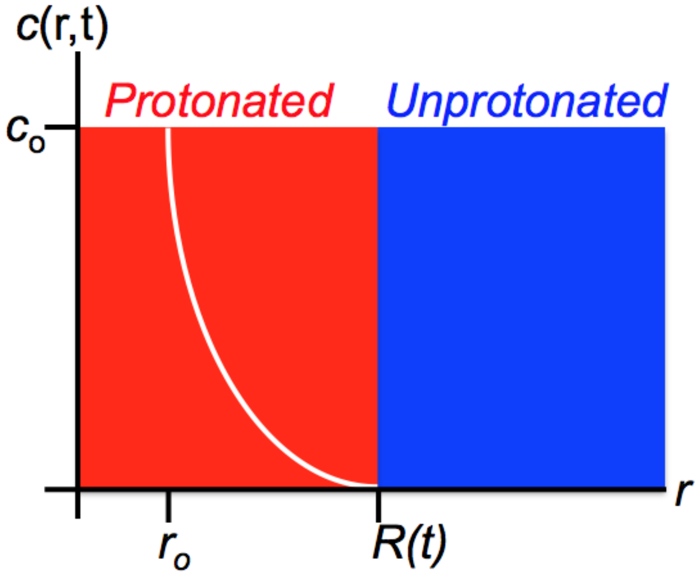

Supplementary Fig. 15 | Diffusion calculation. Sketch of the concentration vs distance profile and associated boundary conditions used for the estimation of the times required to propagate the protonation reaction front over a certain distance.

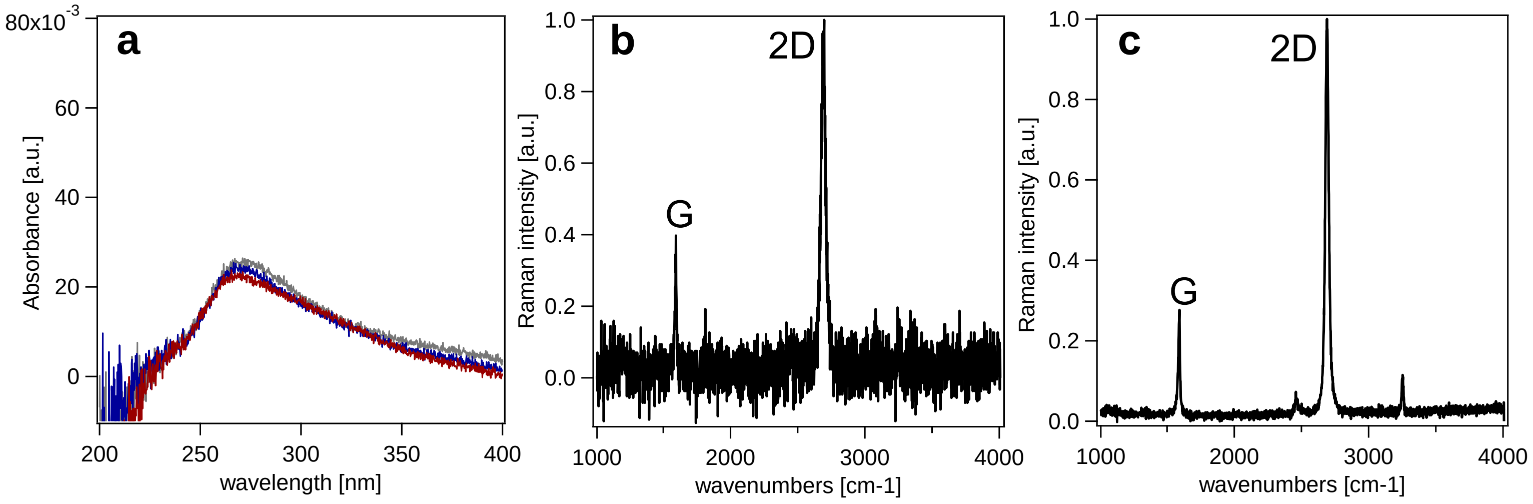

Supplementary Fig. 16 | Stability assessment. (a) Adsorption spectra of a single layer graphene film on a fused silica window before contact with aqueous solutions (grey trace), after a 20 minute soaking in a 1 mM NaCl Millipore water solution adjusted to pH 3 (red trace), and after a subsequent 20 minute soak adjusted to pH 11. Each trace is the average of 7 spectra collected over 7 different spots on the graphene film. Representative Raman spectra of a single layer graphene film **(b)** before SHG “pH jump experiments” and **(c)** after 2 days of SHG “pH jump experiments”. The spectra are normalized to the highest intensity.

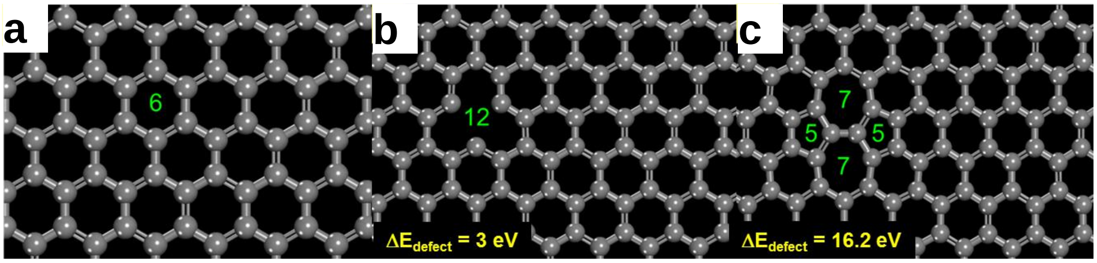

Supplementary Fig. 17 | Defect energetics. DFT-optimized structures for (**a**) pristine graphene, (**b**) 1V defect, and (**c**) Stone-Wales type defect sites and the cost to form the defect sites.

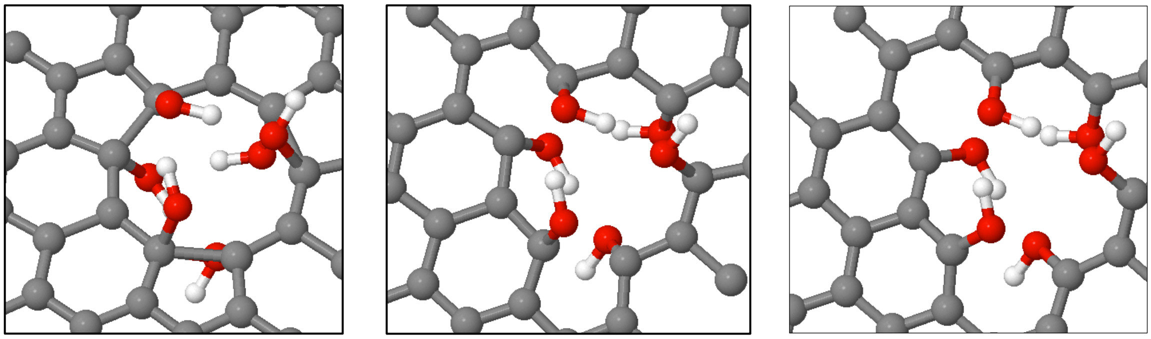

Supplementary Fig. 18 | Relaxation of the defect sites. (from **left** to **right**) The transition from the SW-type defect to the OH-4V-type defect upon hydroxylation: the SW defect was optimized first, followed by putting 6 hydroxyl groups near the defect with the -C-OH bond length being ~1.45 Å (near typical paraffinic C-O bond length). [Denotation: carbon in gray, oxygen in red and hydrogen in white]

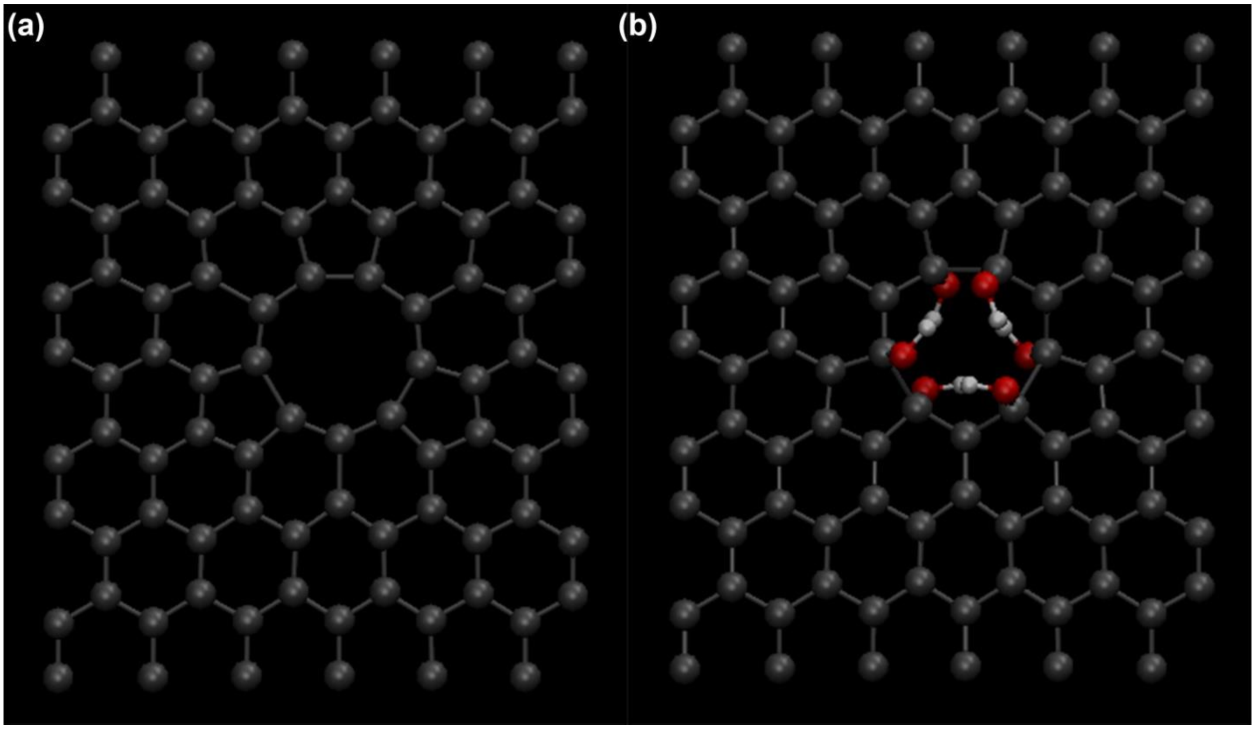

Supplementary Fig. 19. | Defect relaxation and reconstruction. (a) Reconstructed quad-vacancy defect in graphene and (**b**) reconstructed quad-vacancy defect terminated in 6 OH groups from ReaxFF calculations.

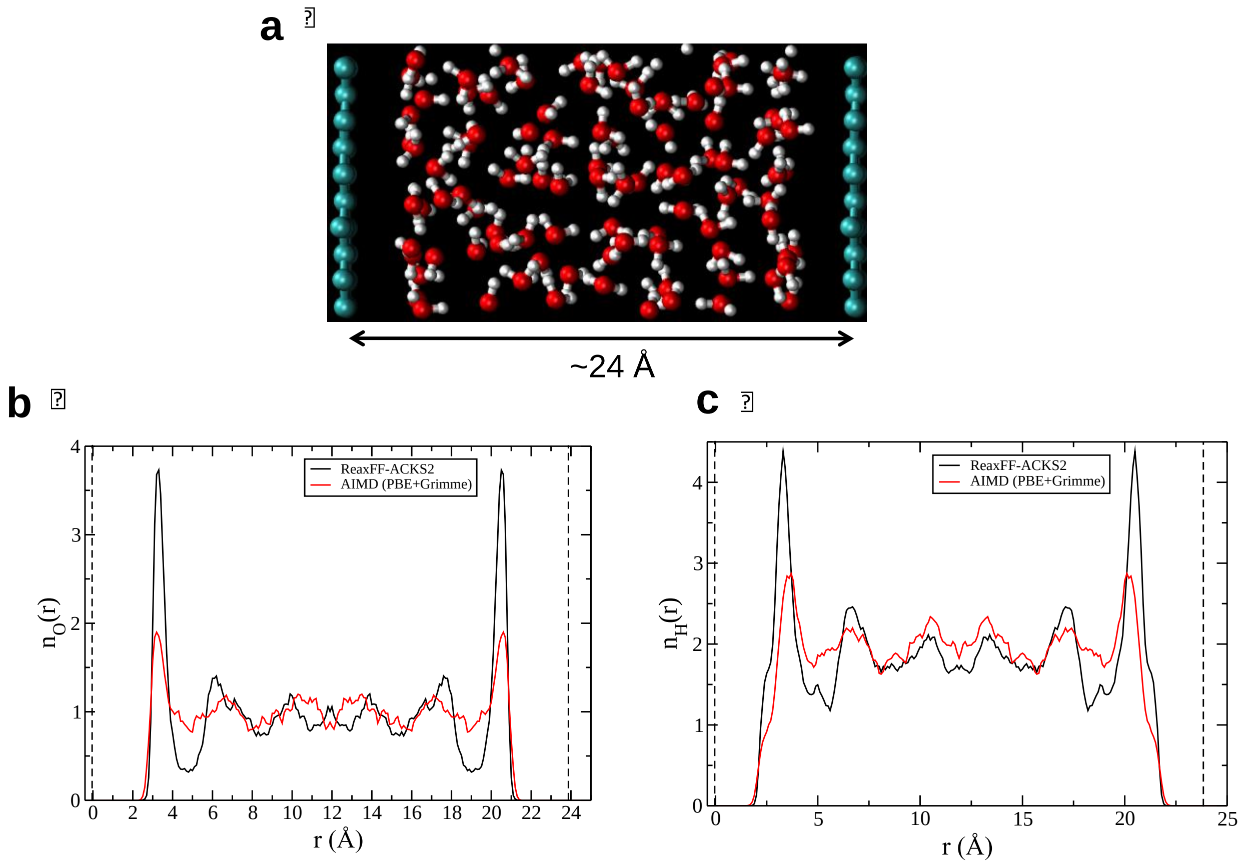

Supplementary Fig. 20 | Structure of water confined between two graphene sheets. (**a**) Graphene/water system used in the ReaxFF/AIMD simulations (**b**) oxygen number density profiles obtained from the ReaxFF/MD and AIMD simulations (**c**) hydrogen density profiles obtained from the ReaxFF/MD and AIMD simulations.

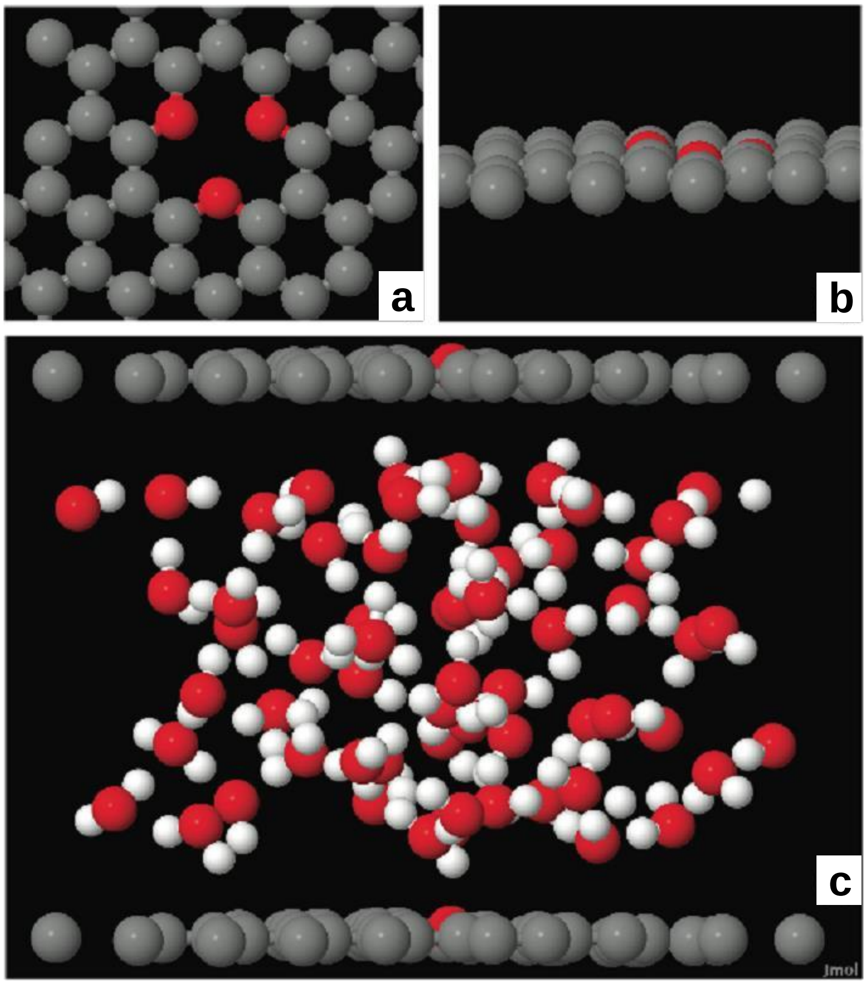

Supplementary Fig. 21 | Ether-like pyrylium terminated defects. The termination of the 4V-defect site in graphene with oxygen can result in the formation of 3 ether-like pyrylium sites. (**a**) top view; (**b**) side view; (**c**) at the water/graphene/water interface. The large grey spheres refer to carbon atoms whereas the, red, and white spheres refer to oxygen and hydrogen, respectively.

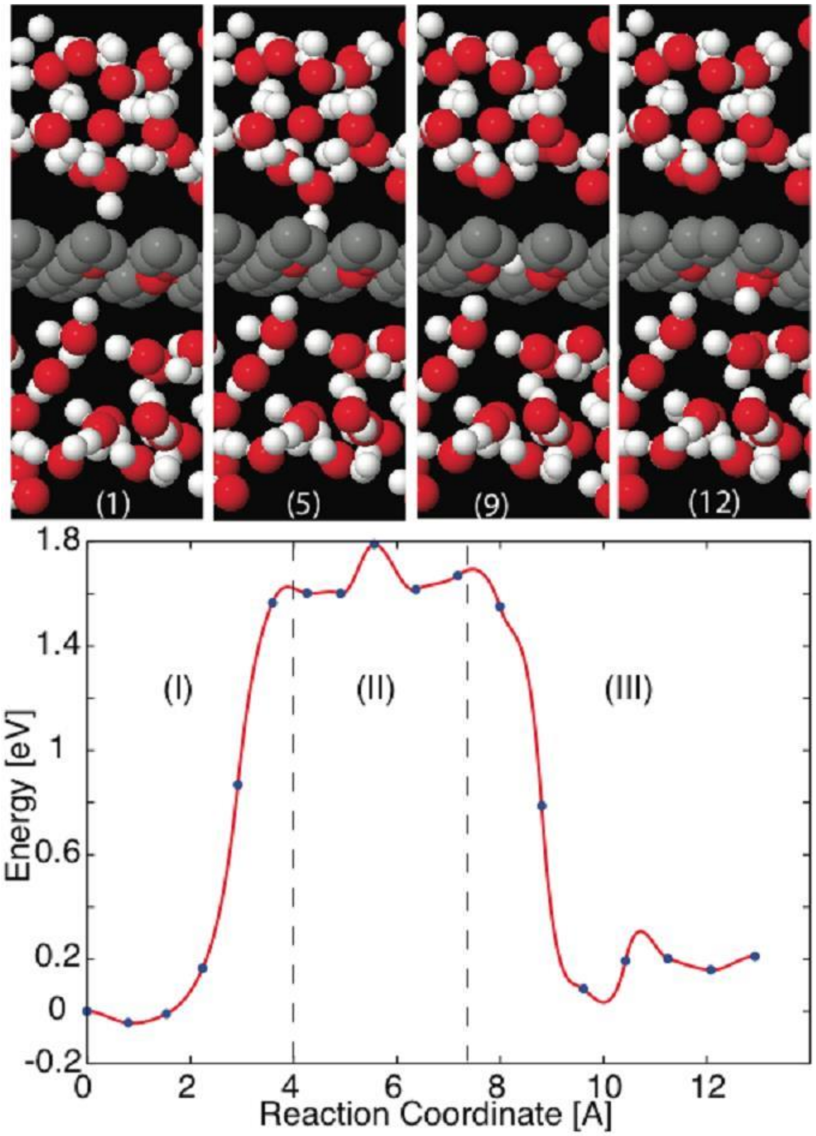

Supplementary Fig. 22 | | Aqueous proton transfer through ether-like pyrylium terminated defects. The reaction energy profile for proton transfer through the ether-decorated pyrylium sites in graphene: (region I) show 1) the release of proton from H_3O^+ to the pyrylium defect site in region II, and 2) relay of proton between ether groups; (region III) release of proton from ether to H_3O^+. [Snapshots from the NEB images are listed as insets on top with the image number marked at the bottom of each inset.]

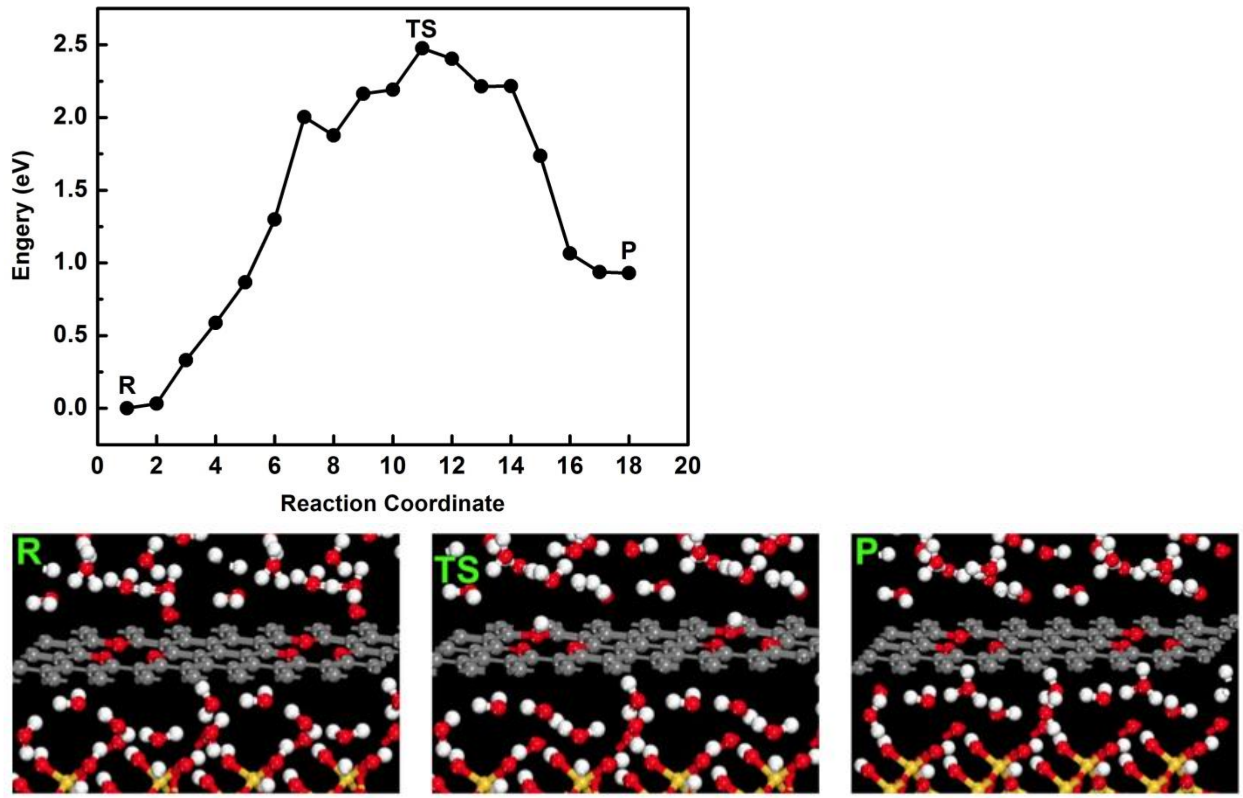

Supplementary Fig. 23 | Proton diffusion through the 3O 4V pyrylium defect site that resides at the water/graphene/water/SiO_2 support interface. The intrinsic barrier was calculated to be 1.8 eV whereas the barrier through the water/graphene/water interface was calculated to be 2.5 eV.

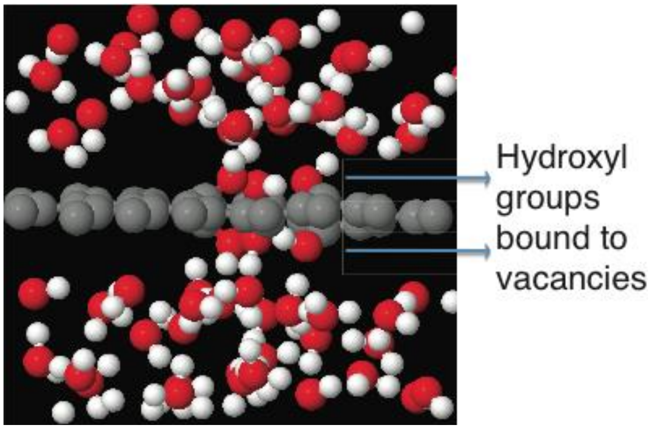

Supplementary Fig. 24 | OH-terminated defect site. The 4V vacancy defect site can react with water to functionalize the 6 coordinatively unsaturated carbon sites in the graphene basal plane with 6 OH groups. Three of the OH groups are oriented into the solution above the graphene surface while 3 are directed into the water solution below the graphene surface. This mixed OH/water interface provides an ideal conduit for proton transfer and proton shuttling.

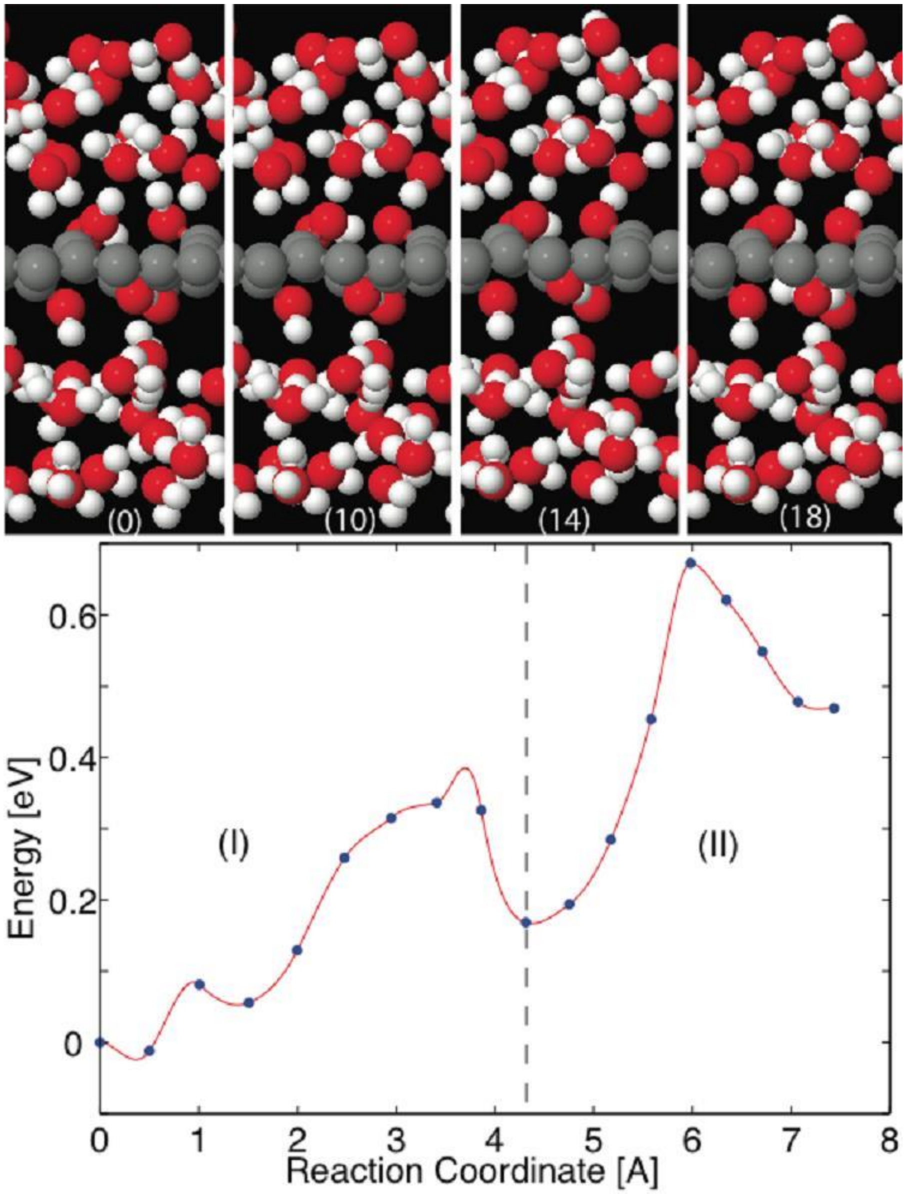

Supplementary Fig. 25 | Aqueous proton transfer across OH-terminated defect site. The energetics for proton transfer through the hydroxyl-decorated graphene: (region I) release of protons from H_3O^+ to hydroxyl/carbonyl groups; (region II) relay of proton between hydroxyl groups on different sides of the graphene sheet; (region III) <not shown here as it is the reverse processes in region I> release of proton from to hydroxyl/carbonyl groups to H_3O^+. Snapshots from the NEB images are listed as insets on top with the image number marked at the bottom of each inset.

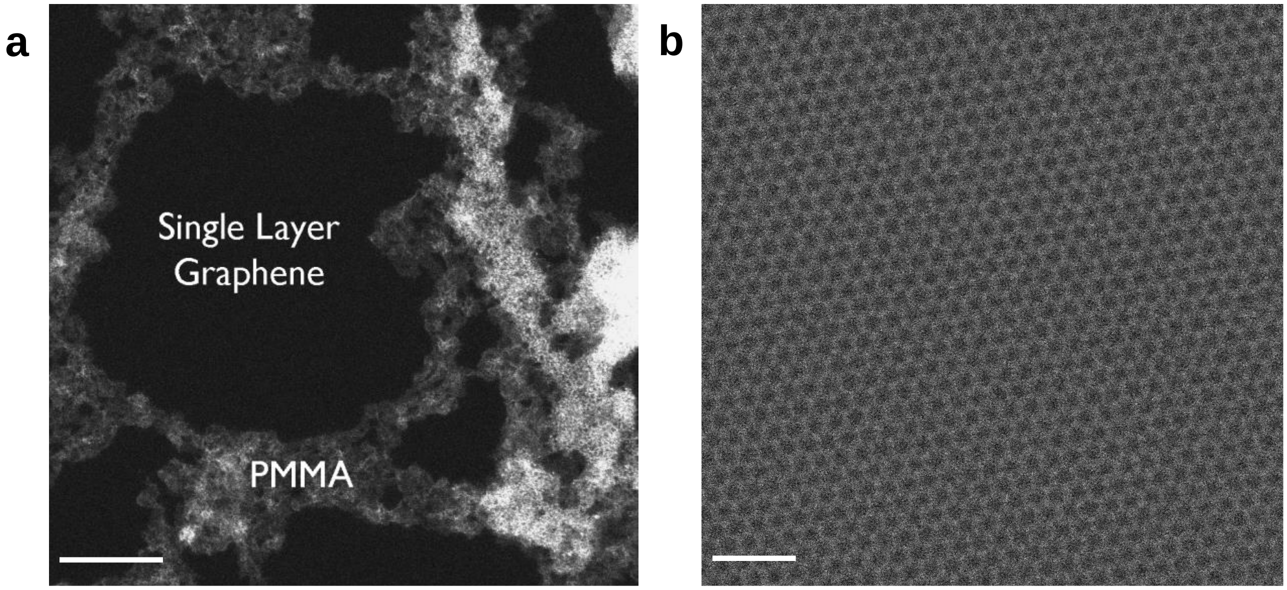

Supplementary Fig. 26 | Imaging pristine graphene. ADF STEM images of CVD prepared graphene showing (**a**) areas of single layer graphene between residual PMMA and (**b**) spatial resolved lattice structure of graphene within the labeled region. Scale bar in (**a**) is 10 nm, and in (**b**) it is 1 nm.

Supplementary Table 1 | Duration of pH jumps as calculated from sigmoid fits. The numbers in parentheses are the standard deviation of the durations.

	**Single layer graphene**		**Fused silica**	
pH jump	3 → 10	10 → 3	3 → 10	10 → 3
jump duration (sec)	22(12)	12(10)	28(19)	14(14)

Supplementary Table 2 | Rates of pH jumps as a function of flow rate.

	**Single layer graphene**				**Bare fused silica**			
pH jump	3 → 10		10 → 3		3 → 10		10 → 3	
flow rate (mL/sec)	0.9	0.3	0.9	0.3	0.9	0.3	0.9	0.3
rate (I_{SHG} counts/sec)	2.5(1)	2.6(4)	1.6(1)	0.7(2)	3.7(1)	2.0 (3)	1.8(1)	0.7(2)
corrected rate (I_{SHG} counts/sec)[a]	1.8(1)	2.7(4)	1.2(1)	0.8(3)	2.6(7)	2.1(3)	1.3(1)	0.7(2)

[a] The resulting rate correcting for relative rate of analyte bulk concentration. Corrected rate values are obtained by correcting for the relative rate of changing ion bulk concentration as reported in ref [17] using the same experimental setup.

Supplementary Table 3 | Calculated probability that the SHG response is due to pinholes.

Time (sec)	Probability
1	3.5 %
2	6.1 %
3	8.2 %
5	12 %
10	21 %

Supplementary Notes

Supplementary Note 1: Analysis of the Magnitude of I_{SHG} Change and the Time Duration of pH Jumps.

As reported previously, SHG intensities are highly sensitive to changes of the interfacial potential, which is produced in the presence of surface charges[1-5]. The SHG response as a function of pH over charged silica surfaces has been studied previously, and it is well understood and accepted that the intensity of the SHG signal is directly related to the population of charged species on the silica surface[1,2,6-8]. Since the point of zero charge (PZC) of fused silica lies between a pH of ~ 2 and 3.5[7,9-16], as the solution pH increases from the PZC pH to pH 10, silanol groups will be deprotonated and the population of negatively charged groups at the surface will increase. In a report by Duval *et al.*[9], the relative surface population of three surface species, $SiOH_2^+$, SiOH, and SiO^-, was quantified using XPS for samples that had been exposed to solutions at pH values ranging between 0 and 10. More specifically, Duval reported silanol group population densities at pH 3 of approximately 7% for $SiOH_2^+$, 83% for SiOH, and 10% for SiO^-

and population densities at pH 10 of approximately 5% for $SiOH_2^+$, 70% for SiOH, and 25% for SiO^-[9]. Thus, with an increase in the solution pH, the SHG signal will increase due to a higher interfacial potential induced from the larger negative surface charge density.

As discussed earlier, the SHG signal in the pH jump experiments is collected as a function of time for a given solution pH value with a constant 1mM NaCl background over both a bare fused silica and a pristine single layer graphene sheet transferred onto a fused silica window. As shown in Fig. 1b in the main text, it is evident that the SHG response of the two systems is very similar. We quantified this similarity by first comparing the relative SHG intensities, I_{SHG}, obtained for the pH jumps for the two systems. To compare the magnitude of I_{SHG} for the two systems, we normalized the SHG E-fields, given by $E_{SHG} = \sqrt{I_{SHG}}$, to the average E_{SHG} at pH 7 from the beginning and end of each pH jump experiment. The averaged SHG E-fields at pH 3, pH 7, and pH 10 for the single layer graphene system and the bare fused silica system are shown in bar graph format in Supplementary Fig.1. This bar graph is comprised of the compiled data for 3 experiments on 2 different graphene samples (blue), and 4 experiments on 2 different silica samples (red). Supplementary Fig. 1 shows that the magnitudes of the normalized SHG E-fields at the three pH values are within error of one another. This similarity indicates that the populations of the charged surface groups for both the graphene and bare fused silica systems are similar.

For our second analysis we examined the duration of the pH jumps between the two systems. As shown in Supplementary Fig. 2 the pH jumps from 3 to 10, and likewise from 10 to 3 both the graphene (blue trace) and bare fused silica (red trace) appear to reach completion within the same time span. In order to quantify the duration of the jumps in both systems, we calculated the time it took for the SHG intensity to increase from 10% to 90%, which was accomplished by

referencing each jump to the average SHG intensity at pH 3, and then normalizing each jump to the average SHG intensity at pH 10. Using IgorPro software, the I_{SHG} vs. time trace was then fit with the following sigmoid:

$$y = b + \frac{n}{1+e^{\frac{(\tau-x)}{r}}} \quad (1)$$

Here, y is the normalized SHG intensity, b is a fit parameter related to the initial I_{SHG} value, n is a fit parameter related to the difference between the initial and final I_{SHG} values, r is the rate by which I_{SHG} changes in time during the jump, τ is the inflection point, and t is time in seconds. We then solved for the corresponding time value at the 0.1 and 0.9 values of the normalized SHG intensity, and averaged the time durations for the 3 to 10, and 10 to 3 jumps respectively for both the graphene and bare fused silica systems. The results for this analysis are summarized in Supplementary Table 1. Considering the time durations calculated here, it is evident that there is no appreciable delay between the graphene and silica systems within the sensitivity of our system.

For our third analysis we compared the rates from the sigmoidal fits for pH jump experiments conducted with flow rates of 0.9 mL s^{-1} and 0.3 mL s^{-1}. The rates for the two pH jumps with both flow rates are summarized in Supplementary Table 2. Following the method described in a previous publication[17], we also reported the rates corrected for the concentration profile of the protons/hydroxides as a function of time. The rates for the pH jumps do not differ between the single layer graphene and bare fused silica systems for either the slow or fast flow rates. Additionally, these results show little appreciable dependence of the rates on the flow rate, which will be important for the diffusion kinetics analysis discussed below. Based on these three analyses we conclude that the two systems do not differ in terms of relative surface charge density, in the duration of the jumps, or in the rates of the jumps, which indicates that the

acid/base chemistry at the fused silica interface occurs in an unimpeded fashion in the presence of graphene.

Supplementary Note 2: Determination of pKa Values by SHG.

To rule out the possibility that the I_{SHG} response over the graphene film is due to proton/hydroxide adsorption above the graphene sheet, the SHG technique was applied to test for the presence of the two well-known acid-base equilibria that have been reported in the literature for fused silica/water interfaces[1,16,18,19]. Specifically, fused silica surfaces contain two types of silanol sites having pKa values ranging between approximately 3.76 and 4.5 for the more acidic sites, and 8.3 and 10.8 for the less acidic sites, depending on the electrolyte identity and concentration[1,16,20]. The existence of these two silanol groups is attributed to two populations of silanol groups involved in either weak or strong hydrogen bond formations with interfacial water molecules[16,20-22]. Experimental evidence for bimodal acid-base equilibria can confirm that the observed SHG response with jumps in bulk pH is due to the protonation and deprotonation of silica surface sites.

The SHG pKa experiments were run on the same experimental setup as described for the SHG pH jump experiments, except that all aqueous solutions contained a 100 mM NaCl background electrolyte solution. The SHG intensity was then collected as a function of pH starting from pH 11 and ending at pH 2.75. As with the pH jump experiments, the desired pH was adjusted and maintained throughout the duration of the experiment with minimum amounts of dilute solutions of ~1M NaOH and HCl. For each pH evaluated, the SHG intensity was allowed to reach steady state at which point the SHG signal was collected for a duration of at minimum 300 seconds. The resulting normalized SHG E-field is plotted as a function of solution pH in Supplementary Fig.

3A. The averaged E-field for each pH is normalized to the averaged E-field at pH 7. In Supplementary Fig. 3A, the resulting E_{SHG} values were then referenced to the minimum SHG E-field around pH 3 and then normalized to the maximum SHG E-field around pH 11. The SHG E-field increases as the bulk pH increases, except that there is a plateau of the SHG E-field starting around pH 6 before another sharp rise in the SHG E-field around pH 8. This plateau and the two inflection points around pH 5 and 9 are consistent with the existence of two surface sites and two pKa values.

Using the effective pKa, (pK_a^{eff}), calculations proposed by Azam *et al.* we can roughly approximate the surface pKa values correcting for the background electrolyte concentration. Here the pK_a^{eff} is given by

$$pK_a^{eff} = p(K_a K_{assc}) = pH_{0.5} - pM^+ \qquad (2)$$

where K_a is the acid dissociation constant, K_{assc} is the background electrolyte cation (M^+) association constant, $pH_{0.5}$ is the pH where the normalize SHG E-field is equal to 0.5 for a given type of silanol, and pM^+ is given by $-\log[M^+]$. In Supplementary Fig. 3B and 3C the normalized SHG E-field is plotted as a function of pH for the more acidic and less acidic silanol groups, respectively. Fitting these traces with a sigmoid curve, the inflection points at $pH_{0.5}$ were used to calculate the pK_a^{eff} for the single layer graphene system in the presence of 100 mM NaCl. We calculated pKa^{eff} values of 3.5(1) and 8.3(2), which fall within the reported literature values[16]. The experimental validation of the existence of two silanol sites and their approximate pKa values confirm that the SHG response is due to acid-base chemistry occurring at the silica surface.

Supplementary Note 3: SHG pH Jump Experiment on Multilayer Graphene.

A multilayer graphene film was prepared by growing bilayer graphene films, and then transferring the bilayer graphene films on top of one another to a silica window several times until an approximate 8-layer graphene film was formed. The normalized Raman spectrum for the multilayer graphene film is shown in Supplementary Fig. 4. The Raman spectrum of the multilayer graphene film exhibits the G and 2D bands associated with graphene films, and a small D band at 1350cm^{-1} indicative of defects[23,24]. Raman spectra were collected over four different spots on the graphene film and exhibited an average G:2D band ratio of 3.1(7) and 2D band full width half max of 86(1) cm^{-1}, where the number in parentheses is the standard error on the point estimate. These values are consistent with multilayer graphene[23-30]. We did not attempt to exactly quantify the number of layers in the graphene film as Raman spectroscopy has been reported to only accurately identify the number of graphene sheets between 1 and 5[23].

The SHG pH jump experiment was then carried on the multilayer graphene. A plot of the SHG intensity as a function pH and time is shown in Supplementary Fig. 5. As with the single layer graphene pH jump results, we see that there is no delay or attenuation of the SHG response with the multilayer graphene (black trace) when compared to the bare fused silica (crimson trace) and the single layer graphene (blue trace). Since the multilayer graphene film was prepared by sequential deposition of ~ double layer graphene films we propose that the quick diffusion process through the multilayer film may be due to proton diffusion though a brick-and-mortar-like structure of the multilayer graphene film, similar to recent work on unimpeded water permeation through graphene oxide films[31]. A brick-and-mortar structure could be formed due to the likelihood of grain boundaries present in the graphene film and the possible water layers that get trapped between the graphene layers during the transfer process.

Supplementary Note 4: Proton Diffusion.

For complex systems with multiple potential proton transfer sites, ReaxFF provides a suitable alternative to the multistate empirical valence bond (MS-EVB) method developed by Voth and coworkers that can successfully describe the proton transport in water and biomolecular systems[32]. For an extremely complicated system with multiple potential reactions such as that under consideration here, the MS-EVB is no longer applicable because this approach requires a combination of states, which represent the chemical bonding topologies involving the proton. In our applications, we have used the ReaxFF method for water/proton/silica systems[33]. In addition, ReaxFF also reproduces experimental results for both water self-diffusion and proton diffusion in water[34]. We therefore employed ReaxFF MD simulations to calculate proton and water self-diffusion constants in water confined between graphene and α-quartz (001) surface. The diffusion constants provide an estimate of the area accessible via proton diffusion during a time *t* from which we compute the probability that the rise and drop of the SHG response over graphene is due to pinholes.

ReaxFF, being a reactive force field, takes chemical reactivity into account, which, as discussed below, is of significance in describing proton diffusion in the presence of surface SiO^- species. The self-diffusion coefficient of water at 298K given by ReaxFF (2.1×10^{-5} cm^2 s^{-1}) is comparable to the values given by diaphragm-cell technique[35] (2.272×10^{-5} cm^2 s^{-1}) and pulsed magnetic field gradient (PFG) NMR (2.299×10^{-5} cm^2 s^{-1}) studies[36]. ReaxFF also reproduces the proton diffusion constant (9.04×10^{-5} cm^2 s^{-1}) in water at 298 K, which is in good agreement with experimental studies[37] (9.31×10^{-5} cm^2 s^{-1}). DFT and ReaxFF calculations predict water

dissociation on a 1V site in graphene to be exothermic with an activation barrier of 47.79 kcal/mol and 36.83 kcal/mol respectively.

The simulated system consists of water molecules sandwiched between graphene and quartz surfaces separated by 1.4 nm as shown in Supplementary Fig. 6. In our simulations we used a periodic quartz (001) slab with (6 x 4) unit cells parallel to the surface with 2 layers of SiO_2 and a periodic graphene sheet with (10 x 5) unit cells parallel to the surface. Water molecules and protons were placed in random configurations between the quartz and graphene surfaces. The dimensions of the simulation cell are 24.73 Å x 13.84 Å parallel to the surface and 25 Å in the direction perpendicular to the surface.

The system was energy minimized with convergence criterion of 0.5 kcal/Å and equilibrated in in the canonical (NPT) ensemble for 100 ps at 300K, with a time step of 0.25 fs using the Berendsen thermostat with a coupling time constant of 100 fs and Berendsen barostat with a coupling time constant of 500 fs to control temperature and pressure of the entire system. We perform MD simulations on the equilibrium configuration for 125 ps at 300K to calculate the proton and water self-diffusion constants. In these simulations the equilibration time was 25 ps and the subsequent production run was 100 ps.

Since the Grotthuss mechanism plays a key role in the proton transport in solution, we evaluated the sequence of proton transport events in our system[38]. To do so, the oxygen (O^*) of the water with an excess proton is distinguished from the neutral water molecules. The same index number of O^* between two adjacent frames from MD trajectory indicates the vehicular transport without proton hopping. A change in the index of the O^* between frames indicates occurrence of Grotthuss-type hopping. Using a time-dependent trajectory of O^*, the diffusion constant of proton transport is calculated based on mean-square displacement and the Einstein relation.

We consider partially hydroxylated (40% SiOH) and fully hydroxylated (100% SiOH) α-quartz(001) surfaces for calculation of the diffusion constants. The self-diffusivity of nano-confined water is reduced roughly by a factor of 24 for the partially hydroxylated (9.537×10^{-7} $cm^2\ s^{-1}$) case and is comparable to liquid water for the fully hydroxylated (2.892×10^{-5} $cm^2\ s^{-1}$) case. Supplementary Fig. 6 gives the density plot of oxygen (blue) and hydrogen (red) atoms in the simulation box during a NVT run at 300 K for (a) partially-hydroxylated and (b) fully-hydroxylated case. As shown in Supplementary Fig. 6, the partially hydroxylated case shows a strongly enhanced 'ice-like' local water structure close to the silica surface whereas the fully hydroxylated case shows a more diffuse water structure similar to liquid water. The O-O separation (2.47 Å) for SiO^-···HOH hydrogen bonds at the partially hydroxylated quartz surface is significantly shorter than O-O separation in bulk water (2.83 Å) indicating the presence of strong hydrogen bonds at the partially hydroxylated quartz/water interface. These strong hydrogen bonds give rise to the 'ice-like' water structure at the partially-hydroxylated quartz/water interface and leads to very low water self-diffusion near the silica surface. The silanol groups on the fully hydroxylated surface form intralayer hydrogen bonds with surface O atoms on the adjacent row in the V-shaped ridges and do not interact with the water phase above, giving rise to a vacuum gap between the fully hydroxylated quartz surface and the water phase as shown in the right panel of Supplementary Fig. 6. This finding explains the absence of a local water structure and faster self-diffusivity at the fully hydroxylated quartz/water interface.

In simulations containing partially hydroxylated quartz surfaces the proton diffusion is quickly terminated by protonation of the surface SiO^- groups. This result indicates that proton diffusion is significantly slower in the presence of surface anionic species due to proton trapping at these sites and the slower water self-diffusion (~factor 24 reduction compared to liquid water). XPS

studies by Duval *et al.* [9] report that surface SiO^- species are present on quartz in the pH range 1 to 10, indicating that proton diffusion is significantly lowered for the entire studied experimental pH range. For the fully hydroxylated case the proton diffusion constant is reduced roughly by a factor of 2 compared to bulk water (4.944 x 10^{-5} cm^2/s).

Supplementary Note 5: SEM Pinhole and Proton Diffusion Analysis.

When considering the pH jumps, it could be possible that the focused 30 μm laser spot is centered directly over a pinhole. In this scenario, the presence of a pinhole could account for the similarity of the SHG response between the bare silica and graphene systems. In this case, the protons could easily pass directly though the pinhole and access the silica surface sites in order to participate in the acid-base chemistry needed to account for the rise and drop in the SHG signal intensities. The probability that our SHG response originates from a pinhole allowing for proton transfer from above the graphene film to the silica surface underneath can be calculated by determining the probability that our SHG beam is focused over a pinhole. However, the probability we need is not simply the percent area of the SEM image covered by pinholes, instead, the area accessible via proton diffusion at time *t* and the area of the focused laser beam also need to be taken into consideration.

For our analysis, SEM images were collected on the graphene film to detect pinholes. The SEM images were collected in a grid-like manner in order to obtain a continuous 529 μm x 397 μm area image at the center of the graphene film. To determine the pinhole density, the images were combined as shown in Supplementary Fig. 7, which is representative of a central area of the graphene film where the SHG experiments are conducted. In order to compensate for the difficulty in identifying pinholes due to charging effects, the image was analyzed by eye, and any

anomaly observed using ImageJ software that was greater than 9 pixels in size was outlined, and then marked as a pinhole by setting the outlined feature to be white (white value=255, 255, 255). For some of these anomalies it was not clear if the imaged feature was a pinhole or a contaminant, such as a piece of dust, debris, or detritus. However, it was decided to treat these features as pinholes and thus compute an upper bound of the pinhole density. Any anomalies smaller than 9 pixels in size were not counted as pinholes as these features are indistinguishable from the image noise. Following thresholding of the brightness to values larger than 254 in ImageJ, we quantified the area that the pinholes covered with the “Analyze Particles” feature in said software. Seven anomalies were detected covering an area of 14 μm^2, or 0.007% of the total image area, as shown in the returned ImageJ “masks” image in Supplementary Fig. 8.

The probability of placing our laser beam within the "diffusion area" emanating from a macroscopic pinhole was then calculated for arbitrarily chosen times of 1 second and 10 seconds by effectively increasing the size of the pinholes on all sides by the proton’s radius of diffusion. The pinholes sizes are graphically increased using Adobe Photoshop while transforming the ImageJ scale bar to the pixel equivalent for Photoshop (5.7 μm $pixel^{-1}$). ImageJ was then used to determine the total area covered by the added pinhole size, which represents the “diffusion detection area”, or the area of the film, which, if probed, could account for the observed SHG response. An example of this alteration is shown in Supplementary Fig. 9 for 1 second duration and a 1 x 10^{-6} cm^2 s^{-1} D value. In this case, the probability that our SHG response is due to proton exchange through pinholes and subsequent diffusion from there to the laser spot is 2.3 %. Lastly, it is also necessary to take into consideration that the laser beam is not completely overlapped with the “diffusion detection area”. In that case, the SHG response may not be significantly altered if diffusion were indeed to be the operative mechanism. The largest standard

deviation of the SHG signal intensity is 15%, and therefore, an overlap of less than 85% should result in a distinct observable change in the SHG signal intensity, adding approximately 2 to 3 μm of the total diameter of the laser spot to the "diffusion detection area". We therefore increased the size of the pinholes on all sides by the distance of the proton diffusion radius plus the 3 μm distance to account for partial overlap of the "diffusion detection area" and the laser spot. Again, using ImageJ, we calculated the percent area covered by the new "diffusion detection/laser spot area". The resulting image for this analysis is shown in Supplementary Fig. 10 (1 second duration, D = 1 x 10^{-6} cm^2 s^{-1}). In this case, the final probability that our SHG is due to pinholes is 3.5 %.

The "masks" image shown in Supplementary Fig. 8 was also used to quantify the average pinhole size and the average distance between pinholes. Using the ImageJ software, the "Analyze Particles" function was used to count and output particle area along with the x and y coordinates of the center of mass for each detected particle. Appropriately accounting for the dimensions of the image and the SEM image scale bar, the average area and distance between pinholes was calculated for the 7 detected pinholes. The average area of the pinholes was calculated to be 2(1) μm^2. The distances from each pinhole to every other pinhole was calculated based on the x and y coordinates provided in the "Results" output of the "Analyze Particles" program. These distances were then averaged together to provide the reported pinhole-to-pinhole distance of 300(160) μm. Here, the number in the parenthesis is the standard error on the point estimate.

Supplementary Note 6: ReaxFF Calculations of Heats of Formation for the Considered Ether/Hydroxyl Defect Terminations.

We performed MD-NVT simulations for the various ether/hydroxyl group terminations (3ether, 2ether +2OH, 1ether+4OH, 6OH) considered in this study in the presence of a bulk aqueous phase (96 water molecules) to investigate their relative stabilities at room temperature. We consider a periodic bare quad-vacancy graphene sheet with (10 x 5) unit cells parallel to the surface in contact with a bulk aqueous phase containing 96 water molecules as the reference state. The bare quad-vacancy graphene/water system is considered as the reference state since the graphene sheet utilized in the experimental studies contains defect sites. The various defect terminations were manually prepared by introducing them at the defect site and solvating the excess protons in the aqueous phase. The systems were energy minimized with a convergence criterion of 0.5 kcal/Å and equilibrated in the canonical (NVT) ensemble for 100 ps at 298.15K, with a time step of 0.25 fs using the Berendsen thermostat with a coupling time constant of 100 fs. The total system energy averaged over the final 20 ps of the NVT simulation was used to calculate the heats of formation. Supplementary Fig. 12 gives the heats of formation for the various defect terminations, obtained by considering the bare quad-vacancy graphene/water system as the reference state. The 2ether+2OH termination is the most stable state followed by 3ether, 1ether+4OH and 6OH terminations. All the considered defect terminations are energetically favorable as compared to the bare quad-vacancy system. We note that energy barriers to the formation of the defect sites were not computed.

Supplementary Note 7: Helium and H_2 Diffusion Through Graphene.

Both DFT and ReaxFF simulations indicate that the diffusion of He and H_2 is quite difficult and as such the 4V vacancy sites are essentially exclusive for proton transfer. DFT-calculated activation barriers for He diffusion through the O- and OH- defect sites were found to be greater than 1.8 eV. The larger size of He and its inability to hydrogen-bond prevent it from diffusing through these atomic scale vacancies. The results clearly show that He cannot form hydrogen bonds and thus requires much higher temperatures to overcome activation energies that are > 2.0 eV to diffuse through the 4V sites (see Supplementary Fig. 14 black trace). The results are also consistent with experimental observations and help to rationalize the proton transfer mechanisms, kinetics and sites of interest in graphene.

Our calculations indicate that the DFT-calculated barrier for H_2 to diffuse through the hydroxyl-decorated vacancies sites for the 4V defect of over 3.0 eV (see Supplementary Figure 14 red trace). This is consistent with the results from He and the fact that the larger size of H_2 causes more repulsion, thus resulting in a much higher barrier. Our studies suggest that the proton is the only hydrogen species that can diffuse through the graphene sheet. The H_2 molecule is too large and cannot take advantage of hydrogen bonding.

The results on the size and defect termination requirements for proton, He, and H_2 transfer provide important insights into the properties of graphene and suggest that single layer graphene may be used as a membrane by which to selectively separate protons from a wide range of other species and as such may be useful in various electrochemical processes such as in fuel cells or batteries without any crossover.

Supplementary Note 8: Estimates Regarding the Propagating Reaction Front.

Diffusive transport of protons between the graphene and silica from an atomic defect of radius r_0 can be modeled with a pseudo steady state approximation where the propagation of the reaction front has negligible effect on the proton concentration distribution.[39] We apply radial symmetry and cylindrical coordinates to the general proton concentration, c, vs. distance, r, profile shown in Supplementary Fig. 15. Given the steady-state approximation, we neglect the time derivative in the general diffusion equation. We assume that the instantaneous concentration profile is the steady state solution for the following boundary conditions: $c=c_o$ at $r=r_o$, where c_o is the proton concentration per unit area at the defect site, which has a radius r_o, and c=0 at r=R(t). We then arrive at the following expression: $c(r, t)=c_o \cdot [\ln(ro/R(t))]^{-1} \cdot [\ln(r)-\ln(R(t))]$.

For a diffusion coefficient, D, of protons moving between the graphene and silica, and a given number density of sites that can be protonated, γ, the rate of the propagating protonation front at radius R is given by $dR\ dt^{-1}= D \cdot c_o \cdot [\gamma \cdot R \cdot \ln(R/r_o)]^{-1}$. Solving for t, one arrives at $t = \gamma \cdot (D \cdot c_o)^{-1} \cdot [R^2 4^{-1} \cdot (2 \cdot \ln(R\ r_o^{-1}) - 1) + r_o^2 4^{-1}]$. For a given atomic defect site radius, r_o, of ~ 0.1 nm, a diffusion coefficient, D, of ~ $1 \times 10^{-10}\ m^2\ s^{-1}$, a site density of $\gamma \sim 1\ nm^{-2}$, and a proton concentration at the defect site, c_o, of ~ 6×10^{14} sites m^{-2}, one computes an estimated duration of ~ 1 s for filling an area having a radius R of ~ 100 nm. Slightly larger areas (R=300 nm) take ~ ten seconds to fill. While we caution that these estimates depend on the input values for γ, D, c_o, and r, the results seem reasonable to within a factor of two or three, given the approximations.

Supplementary Note 9: Graphene Characterization and Analysis with Raman and UV-Vis Spectroscopy.

Like previously reported[4,5], Raman and UV-Vis spectroscopies were used as methods to confirm that the graphene films were not altered due to the high and low pH conditions or due to the experimental procedures outlined above. For analysis by UV-Vis spectroscopy, a spectrum of the graphene film was collected prior to solution exposure (Supplementary Fig. 16a, grey trace), after soaking in a 1mM NaCl Millipore water solution at pH 3 for 20 minutes without rinsing (Supplementary Fig. 16a, red trace), and finally after soaking in a 1mM NaCl Millipore water solution at pH 11 for 20 minutes without rinsing (Supplementary Fig. 16a, blue trace). There is no apparent change between the three absorption spectra, confirming that the graphene film can withstand the high and low pH conditions.

For the Raman analysis, spectra were recorded with an Acton TriVista CRS Confocal Raman System using a 514.5 nm excitation wavelength with a 100x objective at a power density < 10^6 W cm^{-2} to avoid sample damage. Raman spectra were collected prior to SHG pH jump experiments and also after 5 consecutive days of SHG experiments, including 2 days of SHG pH jump experiments. The resulting normalized Raman spectra prior and post experiments are shown in Supplementary Fig. 16b and 16c, respectively. An in-depth analysis of the Raman spectra collected from the graphene films prepared for our SHG experiments is available in our previous publications[4,5]. The Raman spectra obtained from our samples are remarkably similar to those recently reported for epitaxially prepared wafer-scale graphene[40]. Moreover, the Raman spectrum collected from samples following pH jumps does not include a D band at 1350 cm^{-1} (which would be indicative of defects), and the G:2D ratio is maintained in the pre- and post-pH jump Raman spectra[23,25]. Like the UV-Vis spectra, there was no appreciable change in the

Raman spectra, confirming that the integrity of the graphene films is maintained at high and low pH conditions, and while subjecting the graphene samples to our experimental procedures, even for several days of SHG experiments.

Supplementary Note 10. Assessment of Diffusion Kinetics in the Experimental Sample Cell. Similar to the analysis in our previous work[17], we wanted to test whether the changes in our SHG response were kinetically controlled rather than mass-transfer limited. First, we compared the duration of the SHG increase/decrease to the bulk diffusion time. To determine whether a concentration gradient would lead to significant bulk to surface diffusion times, we calculated the bulk to surface diffusion time according to

$$\tau_{diff} = \frac{\left(\frac{\Gamma_{1/2}}{C_{bulk}}\right)^2}{D_{bulk}} \qquad (3)$$

where τ_{diff} is the diffusion time from the bulk to the surface, $\Gamma_{1/2}$ is the absolute 50% saturation coverage, C_{bulk} is the bulk proton concentration, and D_{bulk} is the bulk proton diffusion coefficient in water[17,41]. The diffusion time, τ_{diff}, as expressed above, is the time that it takes a proton to travel from the edge of the region, where 100% of the protons in solution are depleted in order to achieve 50% saturation surface coverage. As discussed above, approximately 25% of the silanol groups are deprotonated (SiO^-) at pH 10, and approximately 10% of the silanol groups are deprotonated at pH 3[9]. If we assume between 10^{15} and 10^{14} total surface sites cm^{-2} [17,42], then at any point there are between ~ 1×10^{13} and 2.5×10^{14} surface sites that will undergo protonation or deprotonation. Given the bulk diffusion coefficients[43] of OH^-(aq) , 5.30×10^{-5} $cm^2\ s^{-1}$, and H^+(aq), 9.31×10^{-5} $cm^2\ s^{-1}$, we obtain upper limits on the bulk to surface diffusion

times ranging between 0.004 and 13.0 milliseconds, respectively. Under these timescales we can be confident that the processes sampled in our experiments are not diffusion limited.

In a second analysis, we calculated the thickness of the hydrodynamic boundary layer, which is defined the distance from a solid object (in this case, the wall of the flow tubes) to the location where the fluid velocity is 99% that of the bulk velocity[44]. Assuming laminar flow conditions the hydrodynamic boundary layer thickness can be calculated using the following expression

$$\delta = \left(\frac{v}{D}\right)^{-1/3} \sqrt{\frac{vx}{u_0}} \qquad (4)$$

where δ is the boundary layer thickness, v is the kinematic viscosity of water at room temperature (0.009 cm^2 s^{-1}), D is the proton bulk diffusion coefficient, x is the distance from the flow cell entrance to the focus spot of the laser (0.5 cm), and u_0 is the mean stream velocity[17,41]. Using a mean stream velocity of 1.1 cm s^{-1} (~1 cm inner diameter tubing, 0.9 mL s^{-1} flow rate), δ is approximately 130 μm. Since the hydrodynamic boundary layer thickness here is much smaller than a theoretical 1 cm diameter pellet, curvature effects can be neglected and the mass transfer coefficient, k_c, can be expressed as a function of the mass stream velocity alone using the Frössling correlation[44]:

$$k_c = \frac{D}{d_p}\left(2 + 0.6\sqrt{Re}\sqrt[3]{Sc}\right) \qquad (5)$$

Here D is the bulk proton diffusion coefficient, d_p is the diameter of the pellet, *Re* is the Reynolds number ($Re = u_0/vd_p$), and *Sc* is the Schmidt number ($Sc = v/D$)[17,41,44]. In this simplified expression the mass transfer coefficient, k_c, depends on the mean stream velocity, d_p[17]. As shown in Supplementary Table2, we determined that the proton adsorption rate was independent of flow velocity. This assessment agrees with our previous report using the same experimental setup as discussed here, where we determined that the measured ion adsorption rate

and overall SHG intensity was independent of the flow velocity[17]. Given that the mean stream velocity is independent of proton adsorption, we are confident that the acid/base reactions occurring at the fused silica surface are not mass transfer limited.

Supplementary Note 11. Consideration of Vacancy Reconstruction.

The formation of defect sites formed in the absence of water or oxygen during graphene synthesis lead to reconstruction of the carbon structure resulting in a more stabilized Stone-Wales (SW) type defects to eliminate the unsaturated sites as was found by Büttner et al.[45]. Our own DFT simulations carried out in vacuum show similar results in that the most stable defect sites in vacuum are those in which the unsaturated carbon sites rearrange to form 5 member aromatic ring structures to form double bonds to coordinatively saturate the carbon sites (Supplementary Fig. 17).

DFT calculations carried out by us further show that the unsaturated carbon defect sites can form covalent C-O or C-OH bonds in the presence of oxygen or water and as such spontaneously ring open the 5, 7 and 8 member ring structures to form the OH-terminated 4V site discussed in our present work. Specifically, DFT calculations indicate that while the SW type defect is the most stable configuration in the absence of water or oxygen, it ring opens as hydroxyl groups are brought in contact with the carbon atoms of the defect site. The defect site spontaneously ring opens to form the more favorable 4V sites in which 6 OH groups terminate the unsaturated carbon centers (Supplementary Fig. 18). This result suggests that the reconstructed SW-type defect, which is favorable under dry (vacuum) conditions, likely ring opens to form 4V defect in

aqueous environments.

ReaxFF simulations were also carried out to examine the lowest energy states for the quad vacancy (4V) defects under dry conditions upon termination by surface hydroxyl intermediates as is shown in Supplementary Fig. 19a. ReaxFF-based energy-minimization calculations were carried out with a convergence criterion of 0.25 kcal $Å^{-1}$ to obtain the binding energy per carbon atom for the 4V and reconstructed 4V defect (R4V). The binding energy per carbon atom for the 4V-case is 178.45 kcal mol^{-1} and for the R4V case it is 180.20 kcal mol^{-1}. As such, the R4V system is energetically more stable as compared to the 4V system, in agreement with the results from Buttner and co-workers[45].

We proceed to consider 6 hydroxyl (6OH) terminations of the 4V and R4V defects since the defects will be functionalized in presence of water. The binding energy per atom for the 4V+6OH case is 171.03 kcal mol^{-1} and for the R4V+6OH case is 169.18 kcal mol^{-1}. We equilibrated the R4V+6OH system in the NPT ensemble for 25 ps with a time step of 0.25 fs using the Berendsen thermostat with a coupling time constant of 100 fs, and Berendsen barostat with a coupling time constant of 500 fs to control temperature and pressure of the entire system. We observe that the 5-membered rings at the R4V defect open up and the R4V+6OH system relaxes to the unreconstructed 4V+6OH system (Supplementary Fig. 19b). This result implies that upon functionalization of the defect sites, the reconstructed 4V defect relaxes to the unreconstructed 4V case considered in our MD simulations.

Supplementary Note 12. Validation of ReaxFF for Water/Graphene Interfaces.

To validate the ReaxFF graphene/water force field, we compare results from our ReaxFF molecular dynamics simulations of our water on graphene interface (Supplementary Fig. 20a) to long-time *ab initio* molecular dynamics (AIMD) simulations. Supplementary Fig. 20b and 20c show the number density profile of oxygen and hydrogen, comparing AIMD results with our ReaxFF force field. While the strength of the first peak is significantly higher using ReaxFF, the position of the first peak compares very well between the two methods. In addition, the $n_H(r)$ profile shows the presence of a ~1Å hydrophobic gap, in good agreement with AIMD as well as existing experiments.[46,47] The peak is around ~2.4Å from the graphene surface using both AIMD and ReaxFF, suggesting that the water orientation is captured properly using ReaxFF at the interface. Beyond ~7Å, water fluctuates about its bulk density. AIMD was performed using the VASP[48] code and a PBE exchange correlation functional with Grimme parameterization to describe the van der Waals interaction.[49] The AIMD was performed at T=300K, with a time step of 0.5 fs, for 43 ps. ReaxFF molecular dynamics were performed for over 150 ps at T=300K. All simulations are for an NVT ensemble at a density of 1g cm^{-3} with a hundred water molecules.

Supplementary Note 13: Proton diffusion through ether/pyrylium-terminated quad-defect sites and those terminated with OH groups.

The O-terminated defect sites on graphene have been suggested to be in the form of ethers, carbonyls, and lactones [50,51]. The O atom can also sit at the carbon vacancy sites to form cationic aromatic pyrylium ($C_5H_5O^+$) species. DFT optimizations carried out for the O atom at the edge

of the vacancy found that oxygen readily substitutes for C atoms to form an aromatic pyrylium species as is shown in Supplementary Fig. 13 and Supplementary Fig. 21. The O atom sits directly in the graphene plane where the C=O bond and C=C bond lengths are calculated to be 1.33 and 1.37 Å, respectively, characteristic of the aromatic C=O and C=O groups. The charges that were calculated indicate that the C_5H_5O species is positively charged, which is fully consistent with pyrylium cation intermediates.

The three bridging O species that form at the 4V site sit directly within the graphene plane and form pyrylium intermediates (Supplementary Fig. 21). The hydrophobic character of the graphene surface, as well as the cationic charge on the pyrylium species, make it very difficult to transfer protons to the defect oxygen centers. While proton transfer readily occurs in solution phase above and below the graphene surface with barriers of only 0.2 eV, the activation energy required to diffusion through the 3O-terminated 4V defect site was calculated to 1.8 eV (Supplementary Fig. 22 and Table 1) and very likely does not occur at the low and moderate temperatures in this system. Further simulations were carried out to examine proton diffusion through the same 3O pyrylium functionalized defects at the water/graphene/water/SiO_2 interface to examine the role of the underlying SiO_2. The calculated barrier (Supplementary Fig. 23) was found 2.5 eV, which is slightly higher than that for the water/graphene water interface. The SiO_2 structure partially limits the mobility and freedom of the interfacial water, which acts to increase the activation energy.

Hydroxyl terminated vacancy sites can readily form in the presence of water and result in effective hydrogen bonding networks that can stabilize protons and provide efficient conduits for proton transfer. The 6 unsaturated carbon sites in the 4V site react with water to form 6 terminal C-OH groups. The most stable conformation of the 6 OH groups is one in which three of the

hydroxyl groups are oriented into the solution phase above the surface and three are oriented into the solution below the surface (see Supplementary Fig. 24). The strong hydrogen bonds that form between the surface hydroxyl groups and water molecules stabilize protons and provide flexible pathways for proton transfer into solution.

Our DFT calculations show that protons in solution diffuse through the hydroxylated vacancy site with a barrier of only 0.68 eV (see Supplementary Fig. 25 and Table 1). This process can readily proceed at room temperature. The protons are transferred via proton shuttling following a Grotthuss mechanism[52] where the proton in solution take on the form of an H_3O^+ hydronium species which can very efficiently transfer its proton along a network of O-H-O-H bonds that make up the proton relay conduit from the water molecules above the surface through defect OH sites bound to the graphene surface and onto the water molecules below the surface thus resulting in a low activation barrier of only 0.68 eV. This was the most effective proton transfer path found.

Supplementary References

1 Ong, S., Zhao, X. & Eisenthal, K. B. Polarization of water molecules at a charged interface: second harmonic studies of the silica/water interface. *Chem. Phys. Lett.* **191**, 327-335, (1992).

2 Eisenthal, K. B. Second Harmonic Spectroscopy of Aqueous Nano- and Microparticle Interfaces. *Chem. Rev.* **106**, 1462-1477, (2006).

3 Hayes, P. L., Malin, J. N., Jordan, D. S. & Geiger, F. M. Get charged up: Nonlinear optical voltammetry for quantifying the thermodynamics and electrostatics of metal cations at aqueous/oxide interfaces. *Chem. Phys. Lett.* **499**, 183-192, (2010).

4 Achtyl, J. L. *et al.* Free energy relationships in the electrical double layer over single-layer graphene. *J. Am. Chem. Soc.* **135**, 979-981, (2013).

5 Achtyl, J. L. *et al.* Interaction of Magnesium Ions with Pristine Single-Layer and Defected Graphene/Water Interfaces Studied by Second Harmonic Generation. *J. Phys. Chem. B*, (2014).

6 Mifflin, A. L., Gerth, K. A., Weiss, B. M. & Geiger, F. M. Surface studies of chromate binding to fused quartz/water interfaces. *J. Phys. Chem. A* **107**, 6212-6217, (2003).

7 Higgins, S. R. S., A.G.; Knauss, K.G.; Eggleston,C.; Jordan G.; . in *Water-Rock Interactions, Ore Deposits, and Environmental Geochemistry: A Tribute to David A. Crerar* Vol. Special Publication No. 7 (ed R.; Scott Hellman, A.W.;) (The Geochemical Society, 2002).

8 Campen, R. K., Pymer, A. K., Nihonyanagi, S. & Borguet, E. Linking Surface Potential and Deprotonation in Nanoporous Silica: Second Harmonic Generation and Acid/Base Titration. *J. Phys. Chem. C* **114**, 18465-18473, (2010).

9 Duval, Y., Mielczarski, J. A., Pokrovsky, O. S., Mielczarski, E. & Ehrhardt, J. J. Evidence of the Existence of Three Types of Species at the Quartz−Aqueous Solution Interface at pH 0−10: XPS Surface Group Quantification and Surface Complexation Modeling. *The Journal of Physical Chemistry B* **106**, 2937-2945, (2002).

10 Stumm, W. M., James J.;. *Aquatic Chemistry:Chemical Equilibria and Rates in Natural Waters*. (John Wiley & Sons, INC., 1996).

11 Langmuir, D. *Aqueous Environmental Geochemistry*. (Prentice Hall Inc. , 1997).

12 Kosmulski, M. pH-dependent surface charging and points of zero charge: III. Update. *J. Colloid Interface Sci.* **298**, 730-741, (2006).

13 Sverjensky, D. A. & Sahai, N. Theoretical prediction of single-site surface-protonation equilibrium constants for oxides and silicates in water. *Geochim. Cosmochim. Acta* **60**, 3773-3797, (1996).

14 Parks, G. A. Surface energy and adsorption at mineral/water interfaces; an introduction. *Reviews in Mineralogy and Geochemistry* **23**, 133-175, (1990).

15 Parks, G. A. The Isoelectric Points of Solid Oxides, Solid Hydroxides, and Aqueous Hydroxo Complex Systems. *Chem. Rev.* **62**, 177-181, (2002).

16 Azam, M. S., Weeraman, C. N. & Gibbs-Davis, J. M. Specific Cation Effects on the Bimodal Acid–Base Behavior of the Silica/Water Interface. *The Journal of Physical Chemistry Letters* **3**, 1269-1274, (2012).

17 Mifflin, A. L., Gerth, K. A. & Geiger, F. M. Kinetics of chromate adsorption and desorption at fused quartz/water interfaces studied by second harmonic generation. *J. Phys. Chem. A* **107**, 9620-9627, (2003).

18 Zhao, X., Subrahmanyan, S. & Eisenthal, K. B. Determination of pKa at the air/water interface by second harmonic generation. *Chem. Phys. Lett.* **171**, 558-562, (1990).

19 Zhao, X., Ong, S., Wang, H. & Eisenthal, K. B. New method for determination of surface pKa using second harmonic generation. *Chem. Phys. Lett.* **214**, 203-207, (1993).

20 Leung, K., Nielsen, I. M. B. & Criscenti, L. J. Elucidating the Bimodal Acid−Base Behavior of the Water−Silica Interface from First Principles. *J. Am. Chem. Soc.* **131**, 18358-18365, (2009).

21 Ostroverkhov, V., Waychunas, G. A. & Shen, Y. R. New Information on Water Interfacial Structure Revealed by Phase-Sensitive Surface Spectroscopy. *Phys. Rev. Lett.* **94**, 046102, (2005).

22 Du, Q., Freysz, E. & Shen, Y. R. Vibrational spectra of water molecules at quartz/water interfaces. *Phys. Rev. Lett.* **72**, 238-241, (1994).

23 Ferrari, A. C. M., J.C.; Scardaci, V.; Casiraghi, C.; Lasseri, M.; Mauri, F.; Piscanec, S.; Jiang, D.; Novoselov, K.S.; Roth, S.; Geim, A.K.;. Raman Spectrum of Graphene and Graphene Layers. *Phys. Rev. Lett.* **97**, 187401, (2006).

24 Ferrari, A. C. Raman spectroscopy of graphene and graphite: Disorder, electron–phonon coupling, doping and nonadiabatic effects. *Solid State Commun.* **143**, 47-57, (2007).

25 Graf, D. *et al.* Spatially Resolved Raman Spectroscopy of Single- and Few-Layer Graphene. *Nano Lett.* **7**, 238-242, (2007).

26 Malard, L. M., Pimenta, M. A., Dresselhaus, G. & Dresselhaus, M. S. Raman spectroscopy in graphene. *Physics Reports* **473**, 51-87, (2009).

27 Pimenta, M. A. *et al.* Studying disorder in graphite-based systems by Raman spectroscopy. *PCCP* **9**, 1276-1290, (2007).

28 Rao, C. N. R., Sood, A. K., Subrahmanyam, K. S. & Govindaraj, A. Graphene: The New Two-Dimensional Nanomaterial. *Angew. Chem. Int. Ed.* **48**, 7752-7777, (2009).

29 Choi, P., Jalani, N. H. & Datta, R. Thermodynamics and Proton Transport in Nafion: II. Proton Diffusion Mechanisms and Conductivity. *J. Electrochem. Soc.* **152**, E123-E130, (2005).

30 Cançado, L. G. *et al.* Quantifying Defects in Graphene via Raman Spectroscopy at Different Excitation Energies. *Nano Lett.* **11**, 3190-3196, (2011).

31 Nair, R. R., Wu, H. A., Jayaram, P. N., Grigorieva, I. V. & Geim, A. K. Unimpeded Permeation of Water Through Helium-Leak–Tight Graphene-Based Membranes. *Science* **335**, 442-444, (2012).

32 Voth, G. A. Computer Simulation of Proton Solvation and Transport in Aqueous and Biomolecular Systems. *Account of Chemical Research* **39**, 143, (2006).

33 Fogarty, J. C., Aktulga, H. M., Grama, A. Y., van Duin, A. C. T. & Pandit, S. A. A reactive molecular dynamics simulation of the silica-water interface. *Journal of Chemical Physics* **132**, 174704/174701-174704/174710, (2010).

34 van Duin, A. C. T., Zou, C., Joshi, K., Bryantsev, V. S. & Goddard, W. A. *A ReaxFF reactive force field for proton transfer reactions in bulk water and its applications to heterogeneous catalysis*. Vol. Computational Catalysis 223 (Royal Society of Chemistry, 2013).

35 Mills, R. Self-diffusion in normal and heavy water in the range 1-45.deg. *Journal of Physical Chemistry* **77**, 685-688, (1973).

36 Holz, M., Heil, S. R. & Sacco, A. Temperature-dependent self-diffusion coefficients of water and six selected molecular liquids for calibration in accurate 1H NMR PFG measurements. *Physical Chemistry Chemical Physics* **2**, 4740-4742, (2000).
37 Light, T. S., Licht, S., Bevilacqua, A. C. & Morash, K. R. The Fundamental Conductivity and Resistivity of Water. *Electrochemical and Solid-State Letters* **8**, E16-E19, (2005).
38 Lee, S. H. & Rasaiah, C. J. Proton transfer and the mobilities of the H+ and OH− ions from studies of a dissociating model for water. *Journal of Chemistry and Physics* **135**, 124505.
39 Deen, W. M. *Analysis of Transport Phenomena*. (Oxford University Press, 2011).
40 Lee, J.-H. *et al.* Wafer-Scale Growth of Single-Crystal Monolayer Graphene on Reusable Hydrogen-Terminated Germanium. *Science, in press*, (2014).
41 Jung, L. S. & Campbell, C. T. Sticking Probabilities in Adsorption of Alkanethiols from Liquid Ethanol Solution onto Gold. *The Journal of Physical Chemistry B* **104**, 11168-11178, (2000).
42 Somorjai, G. A. *Introduction to Surface Chemistry and Catalysis*. (John Wiley & Sons, Inc., 1994).
43 Tuckerman, M. E., Chandra, A. & Marx, D. Structure and Dynamics of OH-(aq). *Acc. Chem. Res.* **39**, 151-158, (2006).
44 Fogler, H. S. *Elements of Chemica Reaction Engineering*. 3rd edn, (Prentice Hall PTR, 1999).
45 Büttner, M., Choudhury, P., Karl Johnson, J. & Yates Jr, J. T. Vacancy clusters as entry ports for cesium intercalation in graphite. *Carbon* **49**, 3937-3952, (2011).
46 Cicero, G., Grossman, J. C., Schwegler, E., Gygi, F. & Galli, G. Water Confined in Nanotubes and between Graphene Sheets: A First Principle Study. *J. Am. Chem. Soc.* **130**, 1871-1878, (2008).
47 Zhou, H. *et al.* Understanding controls on interfacial wetting at epitaxial graphene: Experiment and theory. *Phys. Rev. B* **85**, 035406, (2012).
48 Kresse, G. & Furthmuller, J. Efficient iterative schemes for ab initio total energy calculations using a plane-wave basis set. *Phys. Rev. B* **54**, 11169-11186, (1996).
49 Grimme, S. Semiempirical GGA-Type Density Functional Constructed with a Long-Range Dispersion Correction. *Journal of Computational Chemistry* **27**, 1787-1799, (2006).
50 Yamada, Y. *et al.* Subnanometer Vacancy Defects Introduced on Graphene by Oxygen Gas. *J. Am. Chem. Soc.* **136**, 2232-2235, (2014).
51 Larciprete, R., Lacovig, P., Gardonio, S., Baraldi, A. & Lizzit, S. Atomic Oxygen on Graphite: Chemical Characterization and Thermal Reduction. *J. Phys. Chem. C* **116**, 9900-9908, (2012).
52 de Grotthuss, C. J. T. Sur la décomposition de l'eau et des corps qu'elle tient en dissolution à l'aide de l'électricité galvanique. *Ann. Chim.* **58**, 54-73, (1806).
